# Obestatin Treatment Counteracts Muscle Wasting by Reactivation of Autophagy in Duchenne Muscular Dystrophy

**DOI:** 10.1002/mco2.70563

**Published:** 2026-01-14

**Authors:** Icía Santos‐Zas, Silvia Costas‐Abalde, Andrea C. Lodeiro, Fátima Fernández‐Barreiro, Tania Cid‐Díaz, Saúl Leal‐López, Jessica González‐Sánchez, Mar García‐Lamela, Lucía Debasa‐Corral, Carlos S. Mosteiro, Kamel Mamchaoui, Vincent Mouly, Xesús Casabiell, Rosalía Gallego, José Luis Relova, Yolanda Pazos, Jesus P. Camiña

**Affiliations:** ^1^ Grupo De Endocrinología Celular Instituto De Investigación Sanitaria De Santiago (IDIS) Complejo Hospitalario Universitario De Santiago (CHUS), Servicio Gallego De Salud (SERGAS), Trav Santiago de Compostela Spain; ^2^ Grupo De Investigación Traslacional En Enfermedades del Aparato Digestivo (GITEAD) IDIS, CHUS, SERGAS, Trav Santiago de Compostela Spain; ^3^ Inserm, Institut de Myologie, Centre de Recherche en Myologie Sorbonne Université Paris France; ^4^ Departamento De Fisiología Universidade De Santiago de Compostela (USC) Santiago de Compostela Spain; ^5^ Departamento De Ciencias Morfológicas USC Santiago de Compostela Spain

**Keywords:** autophagy, Duchenne muscular dystrophy, neural precursor cell expressed developmentally downregulated protein 4, obestatin, ubiquitin‐specific protease 10

## Abstract

The mechanisms by which muscular dystrophy‐related stress is transduced to the autophagic machinery remain poorly characterized. The formulation of strategies should be based on how disruption of these processes results in the deregulation of signaling pathways that contribute to many pathological effects of the disease. In this study, we investigated the molecular mechanism by which the obestatin/GPR39 system, an autocrine signaling with anabolic impact on normal skeletal muscle, restores autophagy in Duchenne muscular dystrophy (DMD). We report that obestatin integrates 5' AMP‐activated protein kinase (AMPK) and mammalian target of rapamycin complex 1 (mTORC1) signaling to control ubiquitin proteasome system (UPS), autophagy–lysosome system, and protein synthesis under dystrophic context. The posttranslational modifications of the E3 ligase NEDD4‐L emerges as the main switch to activate the autophagy in response to obestatin. This includes NEDD4‐L tyrosine phosphorylation and autoubiquitination, which is critical for recruiting the ubiquitin‐specific protease 10 to assemble a deubiquitination complex, that orchestrates the unc‐51 like autophagy activating kinase 1 (ULK1) and class III PI3K (VPS34) complexes. Reactivation of autophagy through obestatin signaling promotes the recovery of physiological skeletal muscle function. Thus, DMD conditions determine permissiveness to the activation of AMPK that sustain autophagy under anabolic conditions stablished by obestatin signaling through mTORC1.

## Graphical abstract

Obestatin signaling reactivates autophagy by NEDD4‐L activation under DMD conditions. Tyrosine switch on NEDD4‐L activates autoubiquitination that serves as a scaffold to recruit USP10 to form a deubiquitination complex, which stabilizes VPS34 to promote autophagy by activation of the Beclin1 complex. In parallel, NEDD4‐L favors AMPK exposure to CaMKKß to activate ULK1. Under DMD conditions, AMPK does not inhibit mTORC1, but sustains ULK1/Beclin1 activity and autophagy. Reactivation of autophagy promotes the recovery of physiological skeletal muscle function. In nonpathological conditions, lack of action of non‐RTK on NEDD4‐L favors interaction between c‐Src and NEDD4‐L, thus impairing the VPS34 stabilization and AMPK activation.

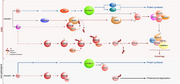

## 1 Introduction

A wide range of rare skeletal muscle disorders are encompassed under the term as myopathy. The most common and detrimental is the Duchenne muscular dystrophy (DMD), which affects about one in 3500 male births [1, 2]. DMD is caused by mutations within the gene encoding for dystrophin, a membrane‐stabilizing protein that plays an essential structural and signaling role both in skeletal and cardiac muscles [3, 4, 5, 6, 7]. In the absence of functional dystrophin, the membrane of skeletal muscle becomes fragile, leading to repeated cycles of tissue degeneration/regeneration, and finally to muscle fiber loss. This condition is further exacerbated by persistent inflammation, fibrosis, dysfunction of autophagy, and tissue necrosis [2, 8, 9, 10]. Despite significant therapy advancements over the past years, a cure for DMD remains elusive [11, 12, 13, 14]. The current standard treatments are corticosteroids, mainly including deflazacort and prednisolone [15]. Both agents exert beneficial effects on the preservation of functional capacities [16, 17]. However, the long‐term benefits and safety of these treatments are uncertain [18, 19]. A number of therapies that target to restore dystrophin or to address secondary pathology have been approved [2]. However, the benefits achieved to date are lower than expected. Therefore, a new therapeutic approach is proposed that requires to improve muscle quality to enhance the effectiveness of treatments aimed at restoring dystrophin [11, 12, 13, 15].

The dystrophin‐associated protein complex (DAPC) was initially considered to be a structural complex that protects the sarcolemma from mechanical damage. However, it is now recognized that DAPC serves as a scaffold for several signaling proteins [20, 21]. As such, DAPC disassembly results in wide‐ranging consequences on muscle cell function [2, 8, 21, 22, 23, 24, 25, 26, 27, 28, 29]. In this sense, the development of strategies should be addressed based on knowledge of the underlying mechanisms associated with DAPC and linked to the pathological development of DMD. Previously, we demonstrated the potential of obestatin, a peptide derived from preproghrelin, and the G‐protein coupled receptor, GPR39 (the obestatin/GPR39 system), as therapeutic target for muscle injury and myopathies related to skeletal muscle regeneration [30, 31, 32]. Obestatin delineates the myogenic program by G‐protein‐dependent and G‐protein‐independent mechanisms linking the activated GPR39 with distinct sets of effector proteins [30, 31, 32]. Added to its function in myogenesis, obestatin activity is associated with muscle remodeling toward an oxidative phenotype and enhancing muscle strength [33]. As an anabolic system, the obestatin/GPR39 system reverses proteostasis, for example, those associated to glucocorticoid‐induced atrophy, and restores efficient basal homoeostasis [34, 35]. Recent studies have demonstrated that the obestatin/GPR39 system restores muscle integrity and function in a DMD murine model [36]. Remarkable, obestatin signaling significantly stabilizes the sarcolemma of skeletal muscle by regulating the expression of utrophin, α‐syntrophin, β‐dystroglycan, and α7β1‐integrin in a DMD model. This observation is associated with decrease of muscle fibrosis, partial rescue of muscle tissue necrosis. All these actions result in substantial improvement in terms of specific force production [36]. In this scenario, the obestatin signaling was unable to increase muscle growth, despite the activation of Akt/mTOR pathway. This fact suggests the existence of other signaling events modulated by dystrophic conditions [36].

Based on this background, in this study, we determined the consequences of the absence of dystrophin on the obestatin/GPR39‐related signaling with special focus on the regulation of autophagy. Using C57BL/10ScSn–Dmdmdx/J (*mdx*) mice at different ages, we show that defective autophagy is related to inverse relationship between mTORC1 and 5′ AMP‐activated protein kinase (AMPK) activity. During ageing, muscle preproghrelin, precursor of obestatin, expression declines while GPR39 protein increases in aged *mdx* mice. Despite being an anabolic system in normal skeletal muscle, we found that, obestatin coactivates mTOR and AMPK signaling to control protein synthesis, the ubiquitin–proteasome system (UPS), and autophagy–lysosome system in *mdx* mouse and human immortalized DMD myotubes. In response to obestatin, NEDD4‐L or NEDD4‐2, an ubiquitin E3 ligase of the NEDD4 family, emerges as the major switch to activate the autophagy machinery. NEDD4‐L undergoes autoubiquitination to act as a scaffold for recruiting the ubiquitin‐specific protease 10 (USP10) to form an NEDD4‐L–USP10 deubiquitination complex. NEDD4‐L orchestrates the Unc‐51‐like kinase 1 (ULK1) and class III PI3K (VPS34) complexes. By reactivating autophagy, obestatin is able to control the establishment of oxidative fibers, to regulate atrophy, and to recover muscle function. Our findings shed light on the mechanisms underlying the temporal coupling between mTOR and autophagy. In this process, amino acids derived from autolysosomes reinforce mTOR activity, and, in this way, counteract muscle wasting in dystrophic conditions.

## 2 Results

### 2.1 Preproghrelin and Autophagy‐Related Changes in *mdx* Mice With Age

**FIGURE 1:**
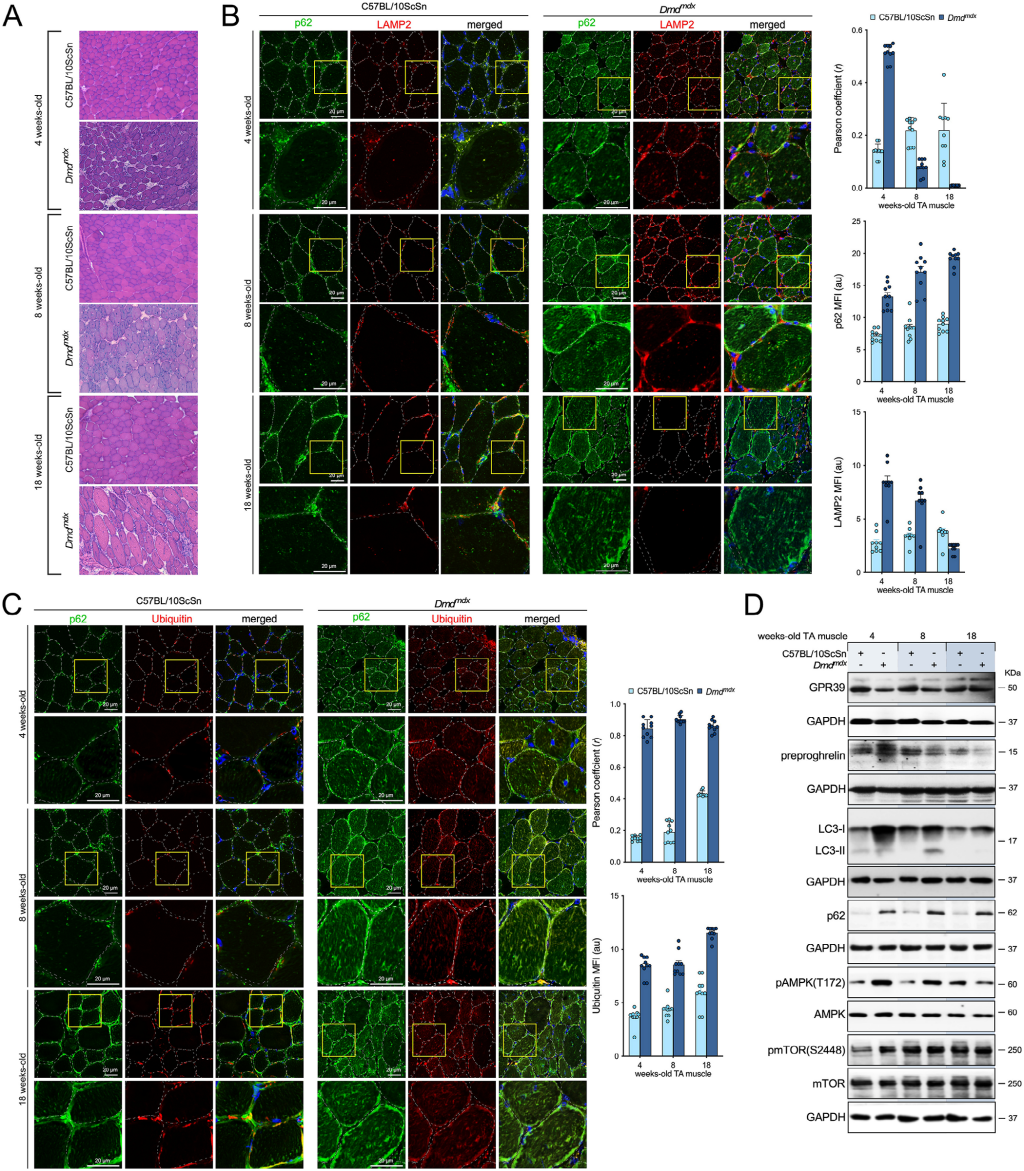
Altered basal autophagy in DMD skeletal muscle. The figure refers to the TA muscles of C57BL/10ScSn and *mdx* mice of different ages (4‐, 8‐, and 18‐week‐old C57BL/10ScSn and *mdx* mice; *n* = 3 per age group). (A) Representative HE staining from TA muscles. (B) *Left panel*: Muscle sections costained for p62 and LAMP2 from the TA muscles. *Right panel*: The changes in MFI of p62 and LAMP2 are shown. Pearson's coefficient (*r*) indicates the correlation of intensity values of green and red pixels in dual‐channel images. Data were expressed as arbitrary units (au). Mean ± SEM; ^*^ *p* < 0.05. (C) *Left panel*: Muscle sections costained for p62 and ubiquitin from the TA muscles. *Right panel*: Pearson's coefficient indicates the correlation of intensity values of green and red pixels in dual‐channel images. Ubiquitin MFI in the TA muscles. Data were expressed as au. Mean ± SEM; ^*^ *p* < 0.05. (D) Immunoblot analysis of GPR39, preproghrelin (obestatin precursor), LC3 (LC3‐I and LC3‐II), p62, pAMPK(T172), AMPK, pmTOR(S2448), and mTOR in the TA muscles.

Our first goal was to achieve histological and molecular characterization of the skeletal muscle of C57BL/10ScSn–Dmdmdx/J (*mdx*) and C57BL/10ScSn (healthy control) mice at different ages. An analysis using hematoxylin and eosin staining (HE) of tibialis anterior (TA) muscles from *mdx* mice at different ages (4‐, 8‐, and 18‐week‐old mice) showed progressive signs of muscle degeneration in *mdx* as compared with control mice. This included early signs of muscle disease from 4‐weeks of age, which progressively worsened. There was evidence of degenerating and regenerating fibers (with centralized nuclei), infiltration of mononucleated cells, and heterogeneity in muscle fiber size variability (Figure 1A). These histopathological features were in line with autophagy‐related changes in *mdx* mice. The accumulation of autophagic vesicles forming from p62 (Figure 1B) and ubiquitin‐positive inclusions (Figure 1C) supported defects in autophagic process in *mdx* mice. The augmented signal of both p62 and ubiquitin and their colocalization suggest a blockage in autophagosomal or lysosomal clearance. Indeed, a significant fraction of p62 positive structures were not labelled by coimmunostaining with lysosomal‐associated membrane protein 2 (LAMP2), a lysosomal transmembrane protein required for fusion of lysosomes with autophagosomes (Figure 1B). We found higher reduction of LAMP2 signal in old with respect to young *mdx* mice (Figure 1B). The enhanced colocalization of p62–ubiquitin aggregates in combination with the deficiency in LAMP2 suggested that the autophagic dysfunction was due, at least in part, to an impairment in lysosomal activity. Compared with young mice (4‐week‐old), aged *mdx* mice (18‐week‐old) showed signs of impaired autophagy flux in basal conditions, with decreased accumulation of the microtubule‐associated protein 1 light chain 3 isoform II (LC3II; membrane‐bound lipidated form) and pronounced augment of p62 (Figure 1D). The impaired autophagy activity was related to sustained, and even increased, mTORC1 activity assessed as phosphorylation of mTOR at S2448 [pmTOR(S2448)] (Figure 1D). The defective autophagy could be explained by the constitutive mTORC1 activation, since DMD‐related stress conditions activates mTORC1 signaling via inactivation of AMPK. Consistent with this notion, AMPK activation [pAMPK(T172)], declined in TA muscles of aged *mdx* mice (Figure 1D). This fact provides a mechanistic explanation for how mTORC1 inhibits AMPK and inactivates autophagy [37]. Contrary to what we might postulate, an inverse correlation between preproghrelin, precursor of obestatin, and GPR39 protein expression was observed with ageing in *mdx* mice (Figure 1D).

### 2.2 Obestatin Modulates Autophagy and Mitochondrial Dynamic in DMD Conditions

**FIGURE 2:**
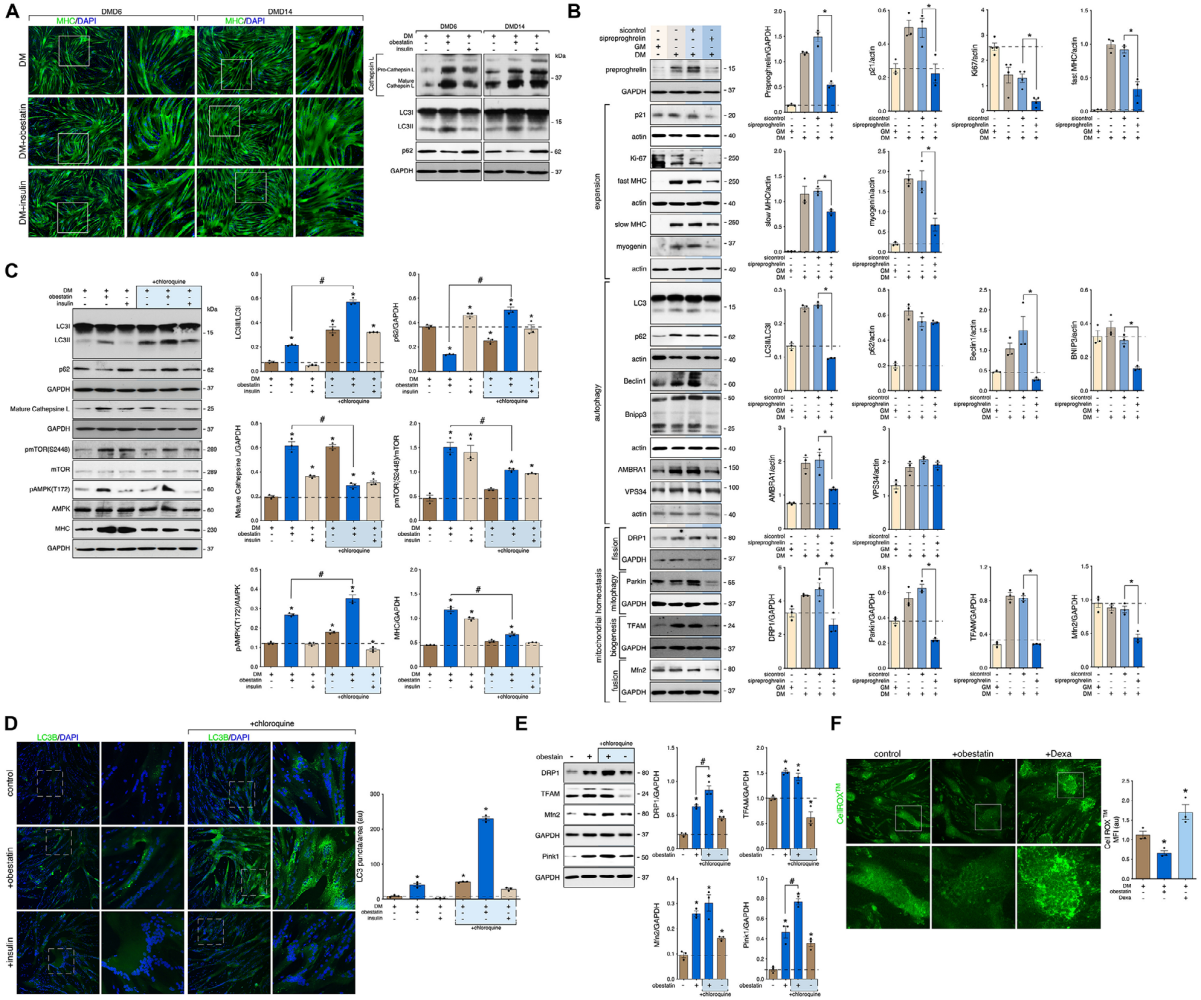
Preproghrelin, and thus obestatin, rescues autophagy flux in DMD cells. (A) *Left panel*: Immunofluorescence detection of MHC in human DMD cell models, DMD6 and DMD14, under DM (control), DM + obestatin (10 nM), or DM + insulin (1.72 µM) 72 h post stimulation. *Right panel*: Immunoblot analysis of p62, LC3, and Cathepsin L, in human DMD6 and DMD14 myotubes at the 72‐h point under DM (control), DM + obestatin (10 nM), or DM + insulin (1.72 µM). (B) DMD myotubes were transfected with siRNA targeting preproghrelin or siRNA‐scramble prior to induction of myogenesis for 96 h. The expression of expansion (p21, Ki‐67, fast‐MHC, slow‐MHC, myogenin), autophagy (p62, LC3, Beclin1, Bnip3, VPS34, AMBRA1) and mitochondrial homeostasis (DRP1, Parkin, TFAM, Mfn2) markers were evaluated by immunoblot analysis (*n* = 3). Immunoblots are representative of the mean value. Data were expressed as the mean ± SEM obtained from intensity scans of independent experiments. ^*^ *p* < 0.05. (C) Immunoblot analysis of p62, LC3, Cathepsin L, pAMPK(T172), AMPK, pmTOR(S2448), mTOR, and MHC in human DMD myotubes at the 24‐h point under DM (control), DM + obestatin (10 nM), or DM + insulin (1.72 µM). Cells were treated with the autophagy inhibitor chloroquine (20 µM) 6 h before collection. Immunoblots are representative of the mean value. Data were expressed as the mean ± SEM obtained from intensity scans of three independent experiments ^*,#^ *p *< 0.05. (D) *Left panel*: Immunofluorescence detection of microtubule‐associated protein 1 light chain 3 isoform B (LC3B) in human DMD cell model under DM (control), DM + obestatin (10 nM), or DM + insulin (1.72 µM) at the 24 h point after stimulation. Cells were treated with the autophagy inhibitor chloroquine. *Right panel*: the changes in LC3 puncta/area from DMD myotube cells are shown. Data were expressed as mean ± SEM (*n* = 3 per group). ^*^ *p* < 0.05. (E) Immunoblot analysis of DRP1, TFAM, Mfn2, and Pink1 in human DMD myotubes at the 24 h point under obestatin treatment (10 nM). Cells were treated with chloroquine before collection. Immunoblots are representative of the mean value. Data were expressed as the mean ± SEM (*n* = 3 per group; ^*,#^ *p* < 0.05). (F) Quantification of ROS levels in DMD myotube cells using CellROX^TM^ fluorescent dye. DMD myotubes were treated with obestatin (10 nM), Dexa (1 µM), or vehicle (control) for 24 h. Results are represented as variation of MFI between control and obestatin‐ or Dexa‐treated cells. Data were expressed as mean ± SEM (*n* = 3 per group). ^*^ *p* < 0.05.

The obestatin/GPR39 system was identified as one of autocrine/paracrine systems involved in the control of mTOR pathways in skeletal muscle [31, 32, 33, 35, 36]. To investigate the involvement of obestatin in autophagy, we used the available in vitro models of human DMD skeletal muscle cells (for details, see section *Material and Methods*). Although being involved in the anabolic system in skeletal muscle (Figure 2A), obestatin treatment upregulated the expression of Cathepsin L and the conversion rate of LC3I to LC3II, with significant reduction in the p62 protein in DMD6 and DMD14, both human DMD cell models. These results were in clear contrast to control or insulin‐treated cells (Figure 2A). Since comparable responses were obtained in both human DMD cell models, DMD14 cells, referred as DMD cells from now on, were chosen for further analyses. The effect of obestatin deficiency was further evaluated by knockdown of preproghrelin with small interfering RNA (siRNA) prior to activation of myogenic differentiation in the human DMD cell model. These conditions decreased the expression of the cell cycle arrest protein p21 and cell proliferation marker Ki‐67 (Figure 2B). The transcription factor myogenin and late marker of differentiation myosin heavy chains (MHCs; slow‐ and fast‐MHCs) were also reduced when preproghrelin was silenced (Figure 2B). Preproghrelin knockdown led to a reduction in the LC3II levels without producing major alterations in levels of p62 (Figure 2B). The positive regulators of autophagy, Beclin1, BCL2 interacting protein (Bnip3), and activating molecule in Beclin1‐regulated autophagy protein 1 (AMBRA1), showed reduced protein expression in sipreproghrelin cells, while no major change was detected in the amount of VPS34 (Figure 2B). We also assessed the effect of sipreproghrelin on factors involved in mitochondrial homeostasis. The protein levels of the fission protein dynamin‐related protein 1 (DRP1), the mitophagy protein Parkin, the biogenesis factor transcription factor A (TFAM), and the mitochondrial fusion factor mitofusin 2 (Mfn2) were decreased in sipreproghrelin cells (Figure 2B). We further confirmed the obestatin‐mediated autophagy reestablishment by using chloroquine. This autophagy‐flux inhibitor impairs the fusion of autophagosome to lysosome, thus increasing LC3II exclusively when autophagy is active. Indeed, chloroquine treatment increased LC3II conversion and p62 accumulation under obestatin treatment, in contrast to insulin‐treated cells (Figure 2C). In this model, the levels of pAMPK(T172) and pmTOR(S2448) were concurrently increased by obestatin, in clear difference to that observe to the insulin response (Figure 2C). Chloroquine treatment was sufficient to inhibit obestatin‐ or insulin‐activated protein synthesis, that is, MHC synthesis, by mTOR inhibition (Figure 2C). Significant accumulation of LC3 immunostaining was detected in obestatin‐treated DMD myotubes (Figure 2D), which was sensitive to chloroquine treatment (Figure 2D). In support of a role in mitochondrial homeostasis, obestatin treatment was further associated with increased DRP1, TFAM, Mfn2, and PTEN‐induced kinase 1 (Pink1) protein levels (Figure 2E). Remarkably, the upregulation of mitochondrial biogenesis‐related proteins was matched by an enhancement of the mitochondrial network (Figure S2A). The presence of myotubes with high values of MitoTracker Deep Red (MTDR) is an indication of correct mitochondrial biogenesis and function. This result is typically associated with gain of mitochondrial membrane potential, or inhibition of apoptosis. Since mitochondria are the primary source of endogenous reactive oxygen species (ROS), we analyzed how obestatin signaling altered ROS [38, 39]. Following obestatin treatment, intracellular ROS levels decreased significantly as evidenced by CellROX Green, a ROS‐sensitive fluorogenic probe. This result contrasted with that achieved with the glucocorticoid agonist dexamethasone (Dexa; Figure 2F). Taken together, these results suggest that preproghrelin, and thus obestatin, is involved in the induction of autophagy in DMD conditions. These data further support a function for obestatin in mitochondrial homeostasis by controlling mitophagy, fission, and biogenesis factors. These observations were associated with enhanced protein synthesis induced by mTOR pathway together with the activation of AMPK signaling.

**FIGURE 3:**
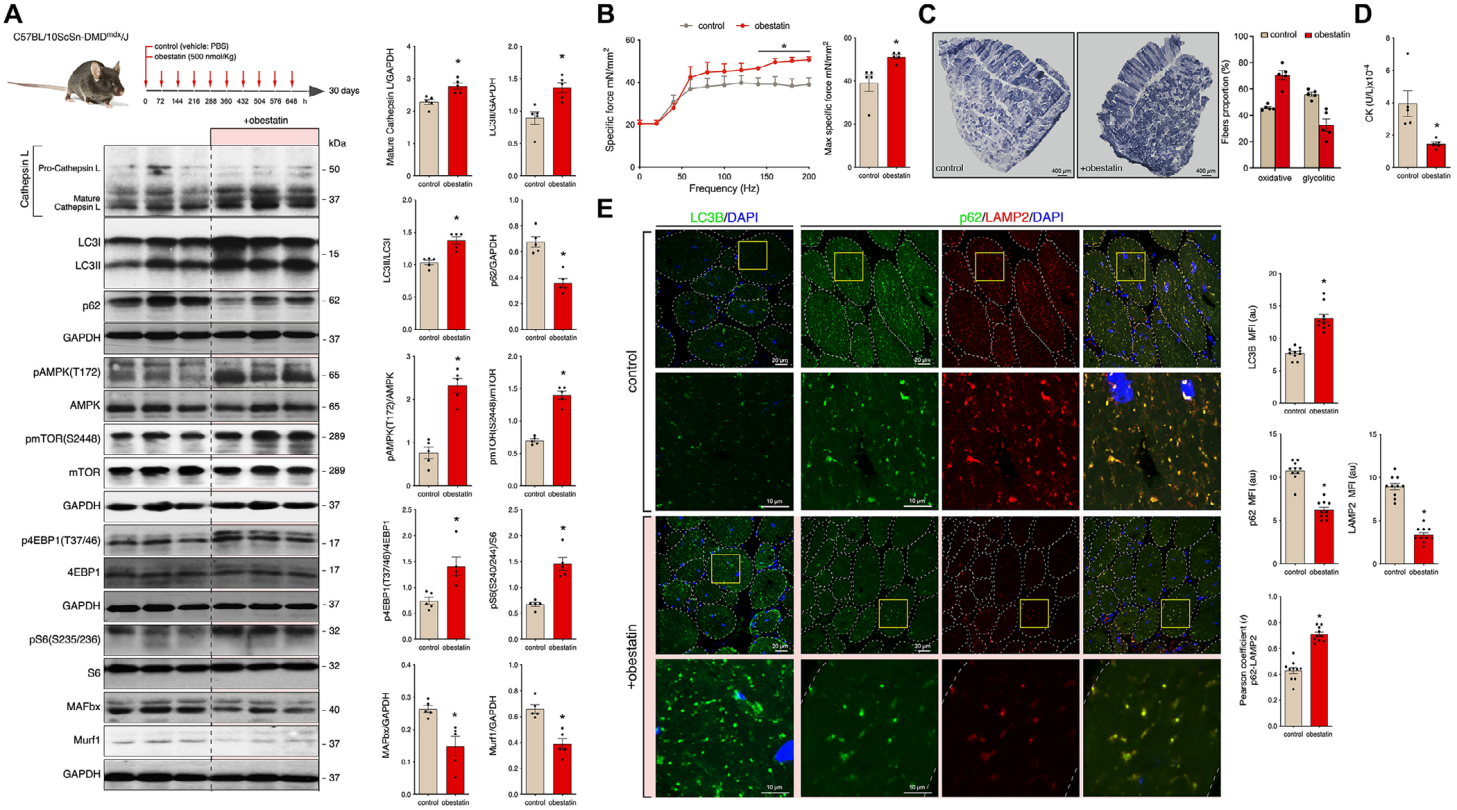
Reinduction of autophagy by obestatin treatment underlies the amelioration of DMD phenotype in *mdx* mice. (A) Effect of intramuscular injection of obestatin (500 nmol/kg body weight each 72 h; *n* = 5) or vehicle (control; *n* = 5) in the TA from *mdx* mice for 30 days. Protein levels of p62, LC3, Cathepsin L, pAMPK(T172), AMPK, pmTOR(S2448) mTOR, p4EBP1(T37/46), 4EBP1, pS6(S240/244), S6, MAFbx, and Murf1 were evaluated by inmunoblot analysis at 30 days. Data were expressed as the mean ± SEM obtained from intensity scans. ^*^ *p* < 0.05. (B) *Left panel*: Force‐frequency curve of TA muscles in obestatin‐treated or vehicle‐treated groups. *Right panel*: Effect of intramuscular injection of obestatin or vehicle on maximum specific force at 30 days. (C) *Left panel*: Representative SDH staining from TA from control muscles and obestatin‐treated muscles at Day 30. *Right panel*: quantitation of oxidative and glycolytic muscle fibers from TA muscles in obestatin‐treated or vehicle‐treated groups. Data were expressed as mean ± SEM. ^*^ *p* < 0.05 versus control values. (D) Serum CK levels after 30 days of treatment with vehicle or obestatin (mean ± SEM; ^*^ *p* < 0.05; *n* = 5 per group). (E) *Left panel*: Representative images of control‐treated and obestatin‐treated TA muscles showing LC3B, p62, and LAMP2 expression. *Right panel*: The changes in MFI of LC3B, p62, and LAMP2 are shown (*n* = 5 per group). Pearson's coefficient (*r*) indicates the correlation of intensity values of p62 (green) and LAMP2 (red) pixels in dual‐channel images. Data were expressed as au (*n* = 5 per group; mean ± SEM; ^*^ *p* < 0.05).

We further tested the effect of obestatin administrated by intramuscular injection into the TA muscles in 8‐week‐old *mdx* mice (for details, see section *Material and Methods*). Tissues were collected at 12 weeks of age, as previously described [36]. These results were compared with those from the vehicle‐treated *mdx* mice (phosphate buffered saline [PBS]; *n* = 5; referred as control group) under identical conditions. Compared with control group, obestatin administration increased the expression of Cathepsin L and the conversion of LC3I to LC3II, with significant decline of p62 protein supporting autophagic activity (Figure 3A). These observations were consistent with concurrent upregulation of pAMPK(T172) and pmTOR(S2448) (Figure 3A). The pmTOR(S2448) was further supported by phosphorylation of the mTORC1 downstream targets 4EBP1 at T37/46 [p4EBP1(T37/46)] and S6 at S235/236 [pS6(S235/236)] (Figure 3A). Notably, obestatin‐treated TA muscles showed lower expression of the E3 ubiquitin‐ligase Murf1 and MAFbx (Figure 3A), in correlation with the improvement of muscle strength (Figure 3B). Obestatin increased the relative density of oxidative fibers (measured as succinate dehydrogenase [SDH] positive fibers [SDH+]), compared with control *mdx* mice (Figure 3C). This activity is in line with the increased levels of peroxisome proliferator‐activated receptor gamma coactivator‐1 alpha (PGC1α), and translocase of outer mitochondrial membrane 20 (TOM20) (Figure S2B). The total amount of mitochondria was increased in obestatin‐treated TA muscle (Figure S2C). These results supported a role in the control of mitochondrial biogenesis. Furthermore, decreased serum creatine kinase (CK) levels, an indicator of muscle damage, was observed in obestatin‐treated *mdx* mice relative to control mice (Figure 3D). Histological examination of the TA muscle showed downregulation of LAMP2 levels in obestatin‐treated *mdx* mice relative to control mice (Figure 3E). LAMP is an indicator of lysosome function, whereby LAMP upregulation corresponds to lysosomal failure [40, 41, 42]. Notably, the dissipation of aggregates forming of p62 and LAMP2‐positive inclusions after obestatin treatment indicated traits of efficient autophagy (Figure 3E). The increased colocalization signal of both p62 and LAMP2 proteins supported that obestatin signaling overcame the autophagic defect, at least in part, by unblocking autolysosomal clearance [42]. Marked increase of LC3 immunofluorescent staining was also observed after obestatin treatment (Figure 3E), which represents autophagic synthesis. These results suggest that obestatin reactivates autophagic flux in dystrophic conditions, and represses the ubiquitin–proteasome‐dependent proteolysis involved in muscle wasting.

**FIGURE 4:**
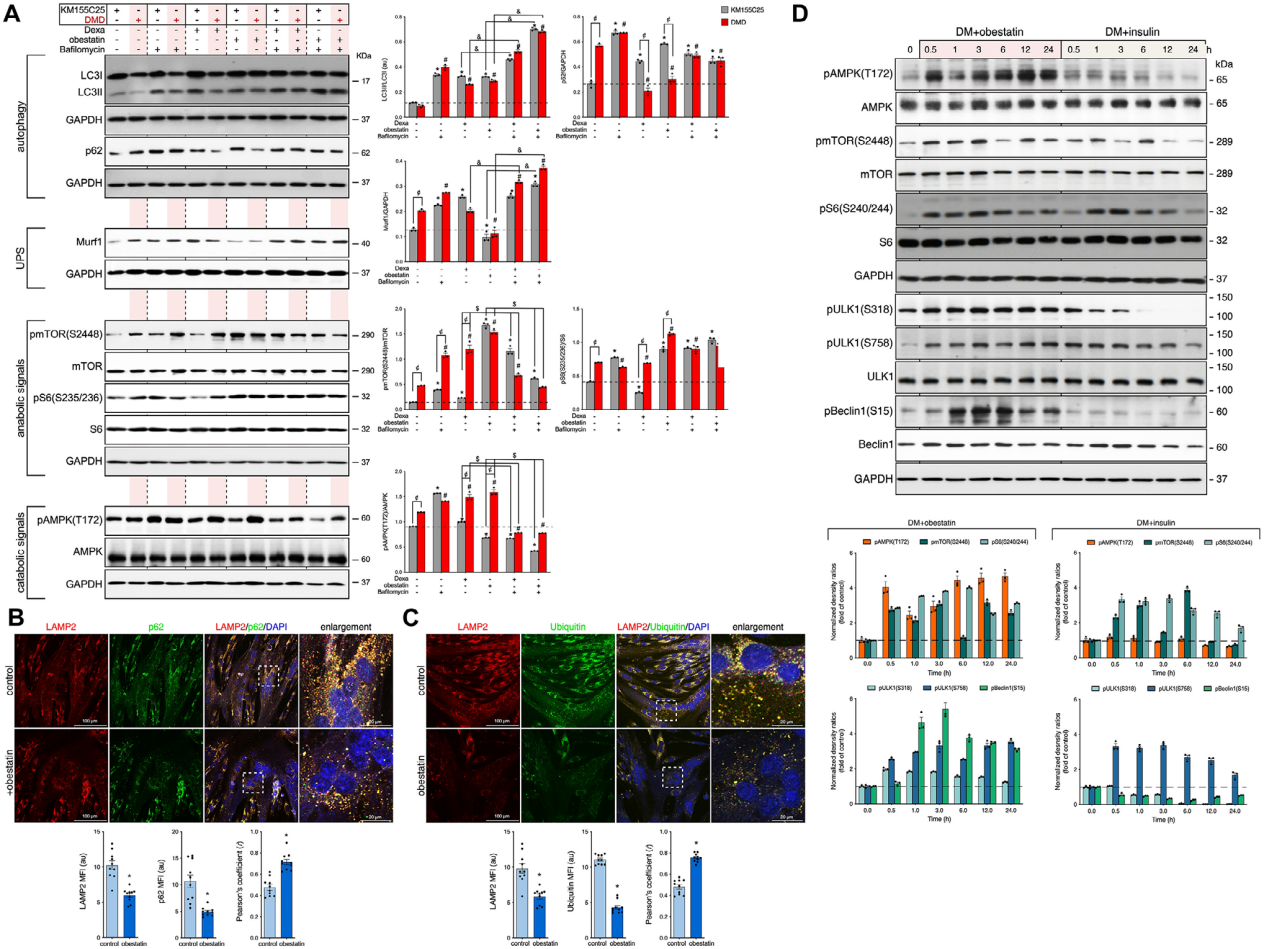
Dystrophic conditions set a different activation pattern for the obestatin/GPR39 system compared with normal human myotubes. (A) KM155C25 and DMD myotubes were treated with obestatin (10 nM) or Dexa (1 µM) in the presence or absence of bafilomycin treatment. LC3, p62, Murf1, pmTOR(S2448), mTOR, pS6(S235/236), S6, pAMPK(T172), and AMPK were analyzed by immunoblotting. Immunoblots are representative of the mean value. Data were expressed as the mean ± SEM obtained from intensity scans from at least three independent experiments (^*,#,&,⊄,$^ *p* < 0.05). (B) *Upper panel*: Representative images from LAMP2 and p62 immunostaining of DMD myotubes treated with obestatin (10 nM) or vehicle (control) for 24 h. *Lower panel*: The changes in MFI of LAMP2 and p62 are shown. Pearson's coefficient (*r*) indicates the correlation of intensity values of green and red pixels in dual‐channel images. Data were expressed as arbitrary units (au) (*n* = 5 per group; mean ± SEM; ^*^ *p* < 0.05). (C) *Upper panel*: Representative images from LAMP2 and ubiquitin immunostaining of DMD myotubes treated with obestatin (10 nM) or vehicle (control) for 24 h. *Lower panel*: The changes in MFI of LAMP2 and ubiquitin are shown. Pearson's coefficient (*r*) indicates the correlation of intensity values of green and red pixels in dual‐channel images. Data were expressed as au (*n* = 5 per group; mean ± SEM; ^*^ *p* < 0.05). (D) Immunoblot analysis of pAMPK(T172), AMPK, pmTOR(S2448), mTOR, pS6(S235/236), S6, pULK1(S318), pULK1(S758), ULK1, pBeclin1(S15), and Beclin1 in full‐time course of DMD myotubes treated with obestatin (10 nM) or insulin (1.72 µM). Protein level was normalized to its nonphosphorylated control (AMPK, mTOR, S6, ULK1, or Beclin1, respectively) and expressed as fold of control (designated as time 0). Data were expressed as the mean ± SEM obtained from intensity scans from at least three independent experiments.

To address how obestatin activates autophagy, we first compared the levels of relevant proteins for autophagy and UPS, along with anabolic and catabolic signals in both DMD and healthy donor‐derived control (KM155C25) myotubes (Figure 4A). In DMD myotubes, obestatin led to an increase in the conversion rate of the LC3I to LC3II with reduced accumulation of p62. Bafilomycin, which prevents fusion between autophagosomes and lysosomes, increased the LC3 conversion and p62 accumulation, confirming the autophagy activation (Figure 4A). Conversely, substantial accumulation of p62 protein was found under obestatin treatment reflecting an autophagy impairment in KM155C25 myotubes (Figure 4A). Obestatin reduced the levels of E3–ubiquitin ligase Murf1 in both DMD and KM155C25 myotubes (Figure 4A). Furthermore, the levels of pAMPK(T172) were increased by obestatin in DMD cells despite pmTOR(S2448) and pS6(235/236) were significantly augmented (Figure 4A). In clear contrast, obestatin abolished pAMPK(T172) in KM155C25 myotubes and increased the levels of pmTOR(S2448) and pS6(235/236) (Figure 4A). Compared with obestatin, Dexa showed similar effect on autophagy in DMD cells, with increased LC3 conversion rate and reduced accumulation of p62 (Figure 4A). In these cells, bafilomycin increased the Dexa‐induced LC3 conversion and p62 accumulation (Figure 4A). By contrast, Dexa showed impaired autophagy flux in KM155C25 myotubes, with marked accumulation of p62 (Figure 4A). Dexa activated AMPK and mTOR phosphorylation in DMD myotubes, without major alterations in the levels of Murf1. Dexa failed to activate mTORC1 and S6 activity in KM155C25 myotubes (Figure 4A). Therefore, we found that both systems acutely activate AMPK concurrently with mTOR to sustain autophagy. Unlike Dexa, obestatin regulates the inhibition of atrophic proteolysis and the activation of homeostatic autophagy in dystrophic conditions. These findings were reinforced by experiments in which DMD myotubes were immunostained for p62, LAMP2 and ubiquitin. The decreased signal of p62 (Figure 4B) and ubiquitin (Figure 4C) and their colocalization with LAMP2 supported that the autophagic defect was overcome, at least in part, by restauration of autolysosomal clearance. When DMD cells were transfected with monomeric red fluorescent protein (mRFP)–enhanced green fluorescent protein (EGFP)–LC3 construct, a tandem fluorescent‐tagged LC3 reporter containing mRFP and EGFP (Figure S3A), obestatin activated the autophagic flux, which was sensitive to bafilomycin treatment (Figure S3B). In the absence of bafilomycin, red LC3 puncta (mature autolysosomes) were abundant in obestatin‐treated cells (Figure S3B,D). In fact, bafilomycin pretreatment blunted this action allowing the accumulation of yellow LC3 puncta (nonfused autophagosomes; Figure S3C). In contrast, red LC3 puncta were rather scarce in obestatin‐treated KM155C25 cells (Figure S3B–D). Thus, obestatin regulates the atrophic proteolysis and the activation of homeostatic autophagy in DMD situation.

### 2.3 Concurrent AMPK and mTOR Activation by Obestatin Signaling in DMD Conditions

AMPK and mTOR are often considered as antagonists that induce or suppress autophagy, respectively [43]. This notion is reinforced by the fact that AMPK phosphorylates and activates tuberous sclerosis proteins 1 and 2 (TSC1/TSC2), thereby inhibiting mTORC1 activity [43, 44]. However, pAMPK(T172) was acutely enhanced by obestatin and this activation apparently did not affect pmTOR(S2448) or mTORC1 downstream target ribosomal protein S6 [pS6(S240/244)] (Figure 4D). Because mTORC1 remains active when AMPK is activated, we examined the implication of ULK1 as autophagic initiator. mTORC1 inhibits autophagy by phosphorylating ULK1 on S758 (corresponding to mouse S757), thereby preventing its interaction with AMPK [45, 46]. On the other hand, AMPK inhibits mTORC1 to prevent S758 phosphorylation on ULK1, leading to interaction and phosphorylation of ULK1 on multiple sites (S318, S467, S555, T575, S637, and S777) to activate ULK1‐dependent autophagy [43, 46, 47]. Interestingly, obestatin signaling enhanced ULK1 phosphorylation at both S318 and S758 (Figure 4D) supporting that ULK1 can be phosphorylated by both mTORC1 and AMPK simultaneously (Figure 4D). Phosphorylation of ULK1 substrate, Beclin1, at S15 [48, 49], was also observed, validating not only the activation of ULK1 but also AMPK (Figure 4D). Thus, AMPK activation by obestatin does not act as a negative regulator of mTOR, which is in clear contrast to other anabolic inputs, such as insulin in DMD myotubes (Figure 4D).

### 2.4 Obestatin Targets AMPK to Activate ULK1 Independently of mTORC1 Activity to Induce Autophagy

**FIGURE 5:**
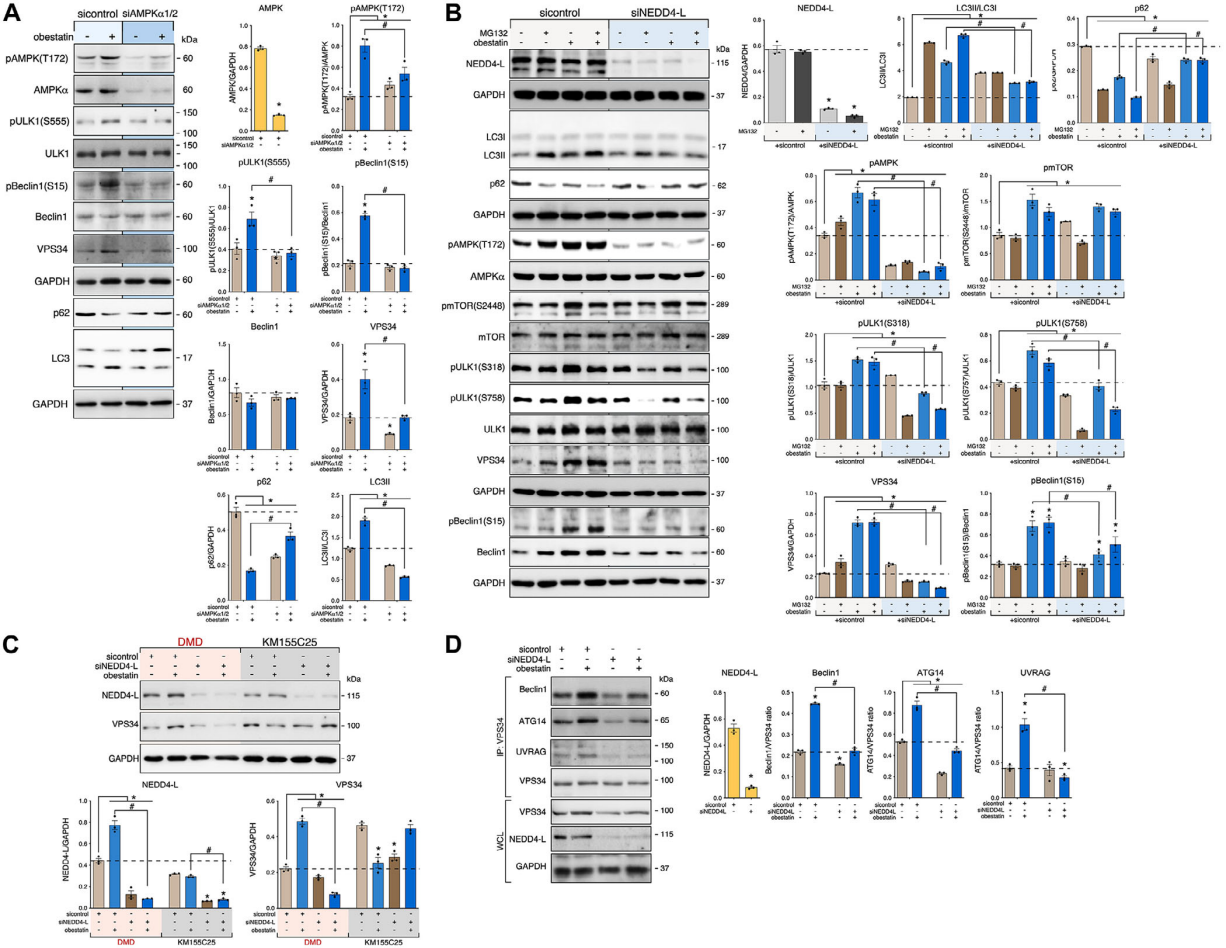
Obestatin stimulates autophagy in an NEDD4‐L‐dependent manner in human DMD myotubes. (A) Immunoblot analysis of pAMPK(T172), AMPK, pBeclin1(S15), Beclin1, VPS34, pULK1(S555), ULK1, LC3, and p62 in DMD myotubes transfected with sicontrol or siAMPKα1/2 and treated with obestatin (10 nM). Data were expressed as the mean ± SEM (*n* = 3 per group; ^*,#^p < 0.05). (B) sicontrol and siNEDD4‐L DMD cells differentiated for 3 days were treated with obestatin in the presence or absence of MG132. Protein extracts were analyzed by immunoblot to detect NEDD4‐L, pAMPK(T172), AMPK, pmTOR(S2448), mTOR, pULK1(S318), pULK1(S758), ULK1, VPS34, pBeclin1(S15), Beclin1, LC3, and p62. Immunoblots are representative of the mean value. Data were expressed as mean ± SEM (*n* = 3 per group; ^*,#^ *p* < 0.05). (C) Immunoblot analysis of VPS34 and NEDD4‐L in extracts of DMD and KM155C25 cells transfected with control or NEDD4‐L siRNAs after obestatin treatment (10 nM). Data were expressed as mean ± SEM (*n* = 3 per group; ^*,#^ *p* < 0.05). (D) Coimmunoprecipitation and immunoblot analysis of extracts of DMD cells transfected with control or NEDD4‐L siRNAs and treated with obestatin (10 nM) by using anti‐VPS34 antibody and immunoblotted with Beclin1, ATG14, UVRAG, VPS34, or NEDD4‐L antibody. Data were expressed as mean ± SEM (*n* = 3 per group; ^*,#^ *p* < 0.05).

We next determined whether AMPK triggers autophagy under mTOR activation. Treatment with Compound C, a known AMPK inhibitor [50], reduced the LC3II levels induced by obestatin favoring accumulation of p62 in DMD cells (Figure S1A). This inhibition was associated with a decrease in AMPK‐dependent ULK1 phosphorylation at both S318 and S555, whereas the mTORC1 substrate site S758 remained unaffected (Figure S1A). Given that Compound C is not particularly specific [50], the effect of AMPKα knockdown by siRNA was assessed (Figure 5A). We observed that AMPKα depletion remarkedly decreased ULK1 phosphorylation at S555 and ULK1 phosphorylation site in Beclin1 (S15) after obestatin treatment. Furthermore, our analysis showed that obestatin increased the protein abundance of VPS34 (Figure 5A). AMPKα depletion resulted in p62 accumulation and reduction of LC3 II thus preventing the activation of autophagy by obestatin in DMD cells. We tested the implication of Ca^2+^/calmodulin‐dependent protein kinase kinase β (CaMKKβ) as AMPK activator [43]. Treatment of DMD cells with STO‐609, a CaMKK β inhibitor [51], reduced the levels of pAMPK(T172) and pULK1(S555), and thus preventing the activation of autophagy by obestatin (Figure S1B). We further examined the autophagy flux in DMD cells in which ULK1 expression was downregulated by specific short hairpin RNA (shULK1; Figure S1C). Under these conditions, DMD cells showed defective autophagy flux as evidenced by reduced LC3II and p62 accumulation (Figure S1D). Thus, obestatin induces autophagy by targeting CaMKKβ/AMPK to activate ULK1 dissociated from mTORC1 activation.

### 2.5 NEDD4‐L–USP10 as Signaling Node to Trigger Autophagy via VPS34 in Response to Obestatin in DMD Conditions

**FIGURE 6:**
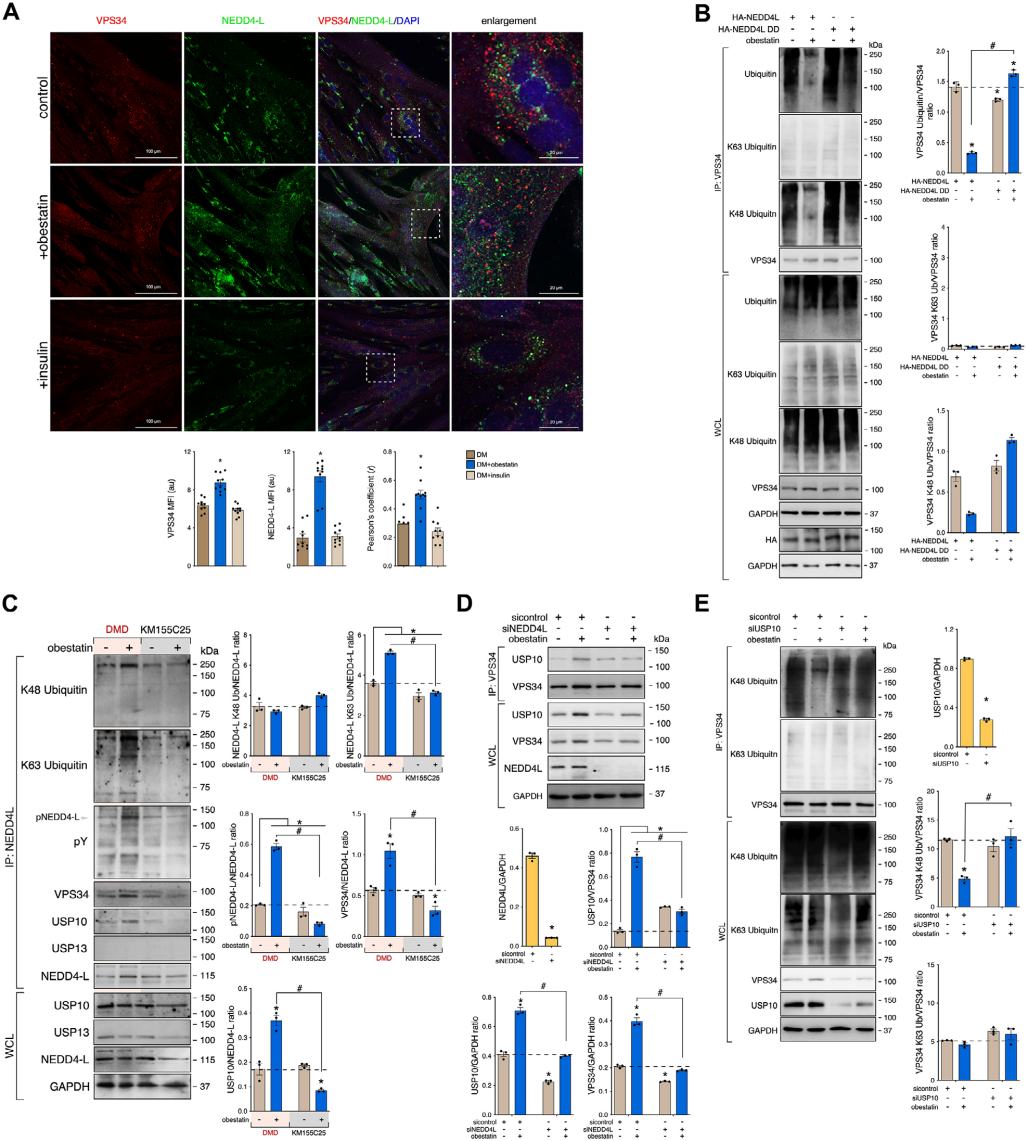
Autoubiquitination of NEDD4‐L facilitate activation of autophagy through the VPS34 stabilization in human DMD myotubes. (A) Representative images from VPS34 and NEDD4‐L immunostaining of DMD myotubes treated with obestatin (10 nM) or insulin (1.72 µM). The changes in MFI of VPS34 and NEDD4‐L are shown. Pearson's coefficient (*r*) indicates the correlation of intensity values of green and red pixels in dual‐channel images. Data were expressed as mean ± SEM (*n* = 5 per group; ^*^ *p* < 0.05). (B) Analysis of VPS34 ubiquitination in DMD cells transfected with HA‐NEDD4‐L or HA‐NEDD4L DD, and treated with obestatin (10 nM, 3 h) in the presence of MG132. VPS34 was immunoprecipitated by using anti‐VPS34 antibody followed by immunoblot analysis with antiubiquitin, antiubiquitin K48 linkage, antiubiquitin K63 linkage, UPS10 or VPS34 antibody. Data were expressed as mean ± SEM (*n* = 3 per group; ^*,#^ *p* < 0.05). (C) Coimmunoprecipitation and immunoblot analysis of extracts of DMD and KM155C25 cells treated with obestatin (10 nM, 3 h) in the presence of MG132 using anti‐NEDD4‐L antibody and immunoblotted with antiubiquitin K48 linkage, antiubiquitin K63 linkage, anti‐pY, anti‐USP10, anti‐USP13, anti‐VPS34, or anti‐NEDD4‐L antibody. Data were expressed as mean ± SEM (*n* = 3 per group; ^*,#^ *p* < 0.05). (D) Coimmunoprecipitation analysis of DMD cells transfected with control or NEDD4‐L siRNAs and treated with obestatin (10 nM, 3 h) by using anti‐VPS34 antibody and immunoblotted with USP10, VPS34, or NEDD4‐L antibody. Data were expressed as mean ± SEM (*n* = 3 per group; ^*,#^p < 0.05). (E) Immunoprecipitation analysis of the VPS34 ubiquitination in DMD cells transfected with control or USP10 siRNAs and treated with obestatin (10 nM, 3 h) in the presence of MG132. Analysis of ubiquitin K48 and K63 linkage was developed by immunoblot. Data were expressed as mean ± SEM (*n* = 3 per group; ^*,#^p < 0.05).

A number of signal transduction kinases have been identified as critical switches of autophagy response [37]. This activity also requires the participation of other posttranslational modifications to control autophagy initiation [52]. Therefore, we explored possible upstream regulators of ULK1 associated to obestatin signaling. First, we evaluated if autophagy initiation was regulated by alternative steps in ULK1, including dephosphorylation by phosphatases and phosphorylation by AMPK, leading to O‐linked N‐acetylglucosamine (O‐GlcNAcylated) on ULK1 at T754 site by O‐linked N‐acetylglucosamine transferase (OGT). ULK1 O‐GlcNAcylation occurs after dephosphorylation of adjacent mTOR‐dependent phosphorylation at S758 site by protein phosphatases (i.e., PP1, PP2A, or PP5) and phosphorylation by AMPK [53, 54]. A coimmunoprecipitation assay against endogenous ULK1 revealed that OGT did not bind to ULK1 upon obestatin treatment in DMD cells (Figure S1D). This is endorsed by the fact that ULK1 O‐GlcNAcylation levels were not altered throughout the course of obestatin treatment (Figure S1D). Although PP1 appeared to interact to some degree with ULK1, this basal interaction was not significantly modified by obestatin treatment (Figure S1D). PP2A and PP5 failed to interact with ULK1 (Figure S1D). We also noticed lack of binding between ULK1 and mTOR during obestatin treatment (Figure S1D). Under normal conditions, obestatin failed to show binding between ULK1 and OGT and subsequent ULK1 O‐GlcNAcylation in KM155C25 cells (Figure S1D). These results ruled out a crosstalk between O‐GlcNAcylation and PPP1‐mediated dephosphorylation directing ULK1 activity in DMD cells. Second, we determined whether tripartite motif family of proteins 32 (TRIM32) interacts with ULK1 and AMBRA1 to stimulate ULK1 activity by favoring their nondegradative ubiquitination [55]. Coimmunoprecipitation of endogenous ULK1 revealed the capacity of TRIM32 to associate with ULK1 in an AMBRA1‐independent manner after obestatin treatment both in DMD and control KM155C25 cells (Figure S1D). Furthermore, obestatin failed to activate ULK1 ubiquitination ruling out the implication of TRIM32 as an E3 ubiquitin ligase that regulates ULK1 activity (Figure S1D). Third, a recent study showed that NEDD4 positively regulates autophagy by editing VPS34 [56]. The role of NEDD4 was evaluated by measuring autophagy flux in DMD cells in which NEDD4‐L expression was reduced by specific siRNA (siNEDD4‐L; Figure 5B). Analysis of LC3II/LC3I conversion rate revealed that obestatin‐activated autophagy flux was defective when NEDD4‐L expression was silenced (Figure 5B). This was endorsed by accumulation of the autophagy adaptor p62 (Figure 5B). Knockdown of NEDD4‐L impaired AMPK phosphorylation at T172 by obestatin treatment despite protein level of AMPK remained unchanged (Figurer 5B). On the contrary, downregulation of NEDD4‐L did not affect the ability of obestatin to phosphorylate mTOR at S2448 but impaired ULK1 phosphorylation at both S318 and S758 (Figure 5B). NEDD4L‐depleted cells exhibited a reduction in the level of VPS34 as well as of Beclin1 (Figure 5B). This action could not be rescued by MG132 (Figure 5B), which shows that NEDD4‐L mediates VPS34 stabilization via deubiquitination. We compared the effects of NEDD4‐L knockdown on VPS34 in both DMD and KM155C25 cells. In contrast to KM155C25 cells, obestatin significantly increased VPS34 expression but NEDD4‐L impairment diminished its level differently in DMD cells (Figure 5C). We next determined whether knockdown of NEDD4‐L would alter the interaction between NEDD4‐L and other components of the VPS34 complex. For this purpose, DMD cells were incubated with obestatin after NEDD4‐L knockdown and VPS34 complex was immunoprecipitated from lysate with VPS34 antibody. The association between VPS34 and other VPS34 complex subunits, including Beclin‐1, ATG14L, and UVRAG, was altered by NEDD4‐L (Figure 5D). Furthermore, confocal microscopy analysis revealed colocalization of VPS34 with NEDD4‐L under obestatin treatment (Figure 6A). To evaluate how NEDD4‐L regulates the stability of VPS34 protein, we examined the ubiquitination of VPS34 after overexpression of NEDD4‐L or the NEDD4‐L (C962A) mutant (NEDD4‐L DD), the catalytically inactive form of NEDD4‐L, in DMD cells (Figure 6B). We found that obestatin decreased the poly‐ubiquitination of VPS34 under NEDD4‐L expression (Figure 6B). By contrast, the NEDD4‐L C962A mutant failed to remove the ubiquitination of VPS34. Furthermore, NEDD4‐L mainly inhibited K48‐linked ubiquitination of VPS34 (Figure 6B). Therefore, NEDD4‐L stabilizes VPS34 via ubiquitin–proteasome pathway.

An important point is to determine how NEDD4‐L mediates the deubiquitination of VPS34. One possibility is that NEDD4‐L serves as a scaffold to assemble a deubiquitination complex that stabilizes VPS34 to initiate autophagy in DMD cells. A coimmunoprecipitation assay against endogenous NEDD4‐L revealed that obestatin increased NEDD4‐L autoubiquitination in DMD cells contrary to what was observed in KM155C25 cells (Figure 6C). Increased NEDD4‐L ubiquitination coincides with NEDD4‐L tyrosine (Y) phosphorylation and VPS34 recruitment, an indication that the activity of NEDD4‐L is increased by obestatin in DMD cells (Figure 6C). Furthermore, obestatin enhanced the association of USP10, but not USP13, as deubiquitinating enzyme to NEDD4‐L in DMD cells contrary to the findings in KM155C25 cells (Figure 6C) [56]. The autoubiquitination of NEDD4‐L appears to be indispensable for the assembly of the NEDD4–USP10 complex, which enables the deubiquitination and stabilization of VPS34 in DMD cells. In fact, depletion of NEDD4‐L by siRNA remarkably decreased the association between VPS34 and USP10 activated by obestatin in DMD cells (Figure 6D). Moreover, knockdown of USP10 by siRNA abolished obestatin‐induced deubiquitination of VPS34 via NEDD4‐L (Figure 6E). These results support the formation of a NEDD4‐L–USP10 complex as essential component for VPS34 deubiquitination under dystrophic conditions.

### 2.6 NEDD4‐L–USP10 Complex as Essential Component for AMPK Deubiquitination in Response to Obestatin in Dystrophic Conditions

**FIGURE 7:**
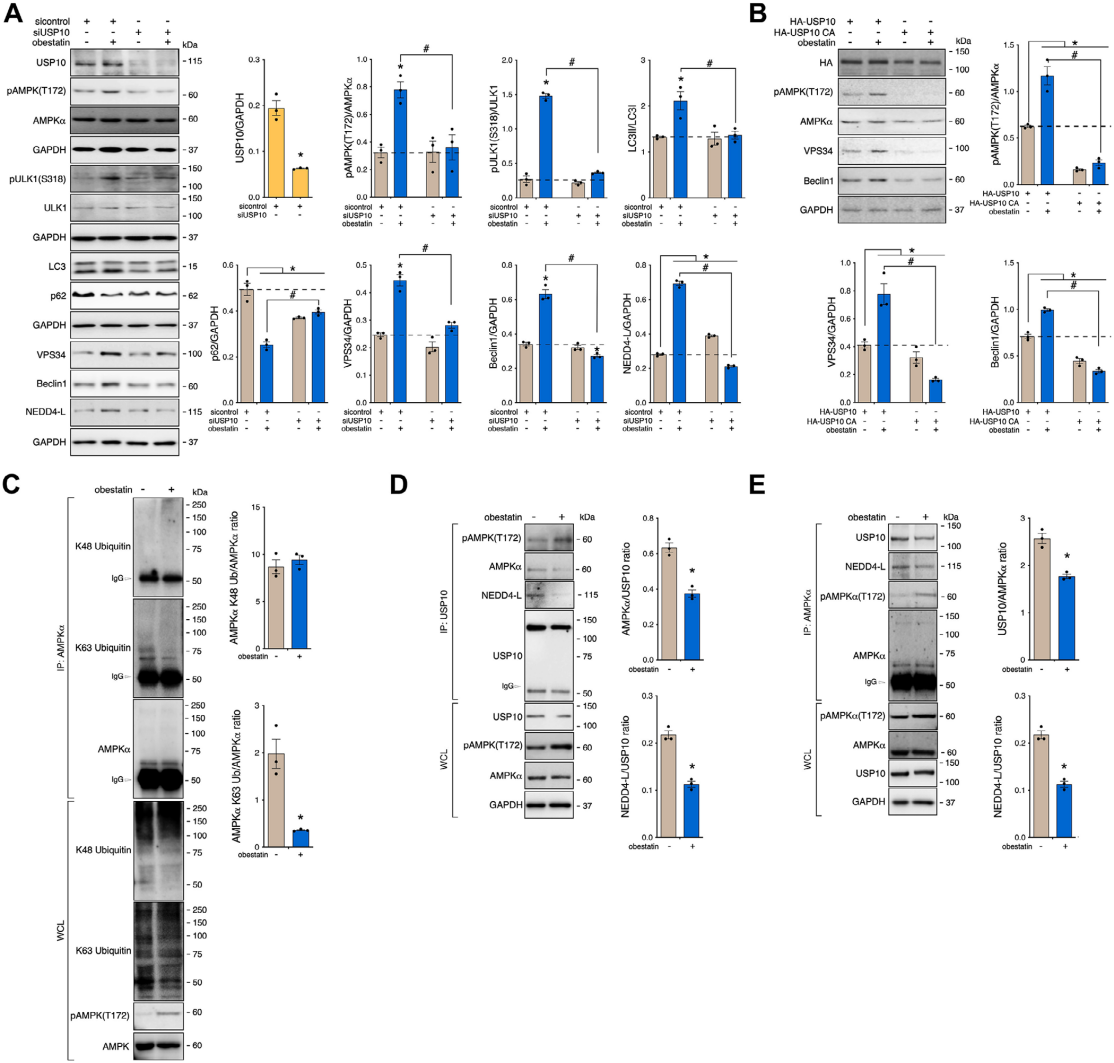
USP10 is required for deubiquitination of VPS34 and AMPKα in human DMD myotubes. (A) Immunoblot analysis of pAMPK(T172), AMPK, pULK1(S318), ULK1, NEDD4‐L, VPS34, Beclin1, LC3, and p62 in DMD myotubes transfected with sicontrol or siUSP10 and treated with obestatin (10 nM, 3 h). Data were expressed as the mean ± SEM (*n* = 3 per group; ^*,#^p < 0.05). (B) Immunoblot analysis of Beclin1, VPS34, pAMPK(T172), and AMPK in DMD cells transfected with HA‐USP10 or HA‐USP10 CA and treated with obestatin (10 nM, 3 h). Data were expressed as the mean ± SEM obtained from intensity scans (*n* = 3; ^*,#^p < 0.05). (C) Immunoprecipitation analysis of the AMPKα ubiquitination in DMD cells after obestatin (10 nM, 20 min) treatment and in the presence of MG132. Analysis of ubiquitin K48 and K63 linkage was developed by immunoblot. Data were expressed as mean ± SEM (*n* = 3 per group; ^*^p < 0.05). (D) Coimmunoprecipitation analysis of DMD cells treated with obestatin (10 nM, 20 min) by using anti‐USP10 antibody and immunoblotted with anti‐pAMPK(T172), anti‐AMPKα, anti‐NEDD4‐L, or anti‐USP10 antibody. Data were expressed as mean ± SEM (*n* = 3 per group; ^*^p < 0.05). (E) Coimmunoprecipitation analysis of DMD cells treated with obestatin (10 nM, 20 min) by using anti‐AMPKα antibody and immunoblotted with anti‐USP10, anti‐NEDD4‐L, anti‐pAMPK(T172), or anti‐AMPKα antibody. Data were expressed as mean ± SEM (*n* = 3 per group; ^*,#^p < 0.05). In panels (A)–(E), immunoblots are representative of the mean value.

As obestatin regulates AMPK activity through NEDD4‐L, we postulated that USP10 would regulate AMPK activation via deubiquitination in DMD cells. When we knocked down USP10 by siRNA, obestatin failed to activate AMPK phosphorylation at T172 despite protein level of AMPK remained unchanged in DMD cells (Figure 7A). Analysis of LC3I to LC3II conversion revealed a defective autophagic flux when USP10 was silenced (Figure 7A). This was endorsed by accumulation of the autophagy adaptor p62 (Figure 7A). In addition, USP10‐depleted cells exhibited a reduction in the level of VPS34 as well as of Beclin1 (Figure 7A). We further overexpressed USP10 or USP10 C424A mutant, the catalytically inactive form of USP10, and found that obestatin activated AMPKα in DMD cells overexpressing USP10, but not the CA mutant (Figure 7B). Only DMD cells overexpressing USP10, but not the CA mutant, were able to stabilize VPS34 and Beclin1 via obestatin (Figure 7B). These results suggest that USP10 is required for AMPK activation. In this context, previous reports demonstrated that USP10 acts as a critical regulator for AMPK activation under energy stress [57]. We then explored the mechanism by which USP10 controls AMPK activation, first by examining changes in the linkage of AMPKα ubiquitination. Immunoprecipitation analysis of AMPKα from DMD cell lysates showed that AMPKα was primarily ubiquitinated by K63‐specific chains under basal conditions, and obestatin decreased the K63–ubiquitin chains (Figure 7E). Using coimmunoprecipitation assay, we found that AMPKα directly interacted with USP10 under basal conditions. However, this interaction was disrupted after obestatin treatment, in correlation with AMPK activation (Figure 7C,D). Interestingly, NEDD4‐L coimmunoprecipitated with USP10 under basal conditions, an interaction also disrupted by obestatin stimulation. Thus, AMPK activation is negatively regulated by ubiquitination and obestatin modulates the NEDD4‐L–USP10 complex to remove the inhibitory ubiquitination of AMPK.

### 2.7 NEDD4‐L Tyrosine Phosphorylation Acts as Scaffold to Form NEDD4‐L–USP10 Complex in Response to Obestatin

**FIGURE 8:**
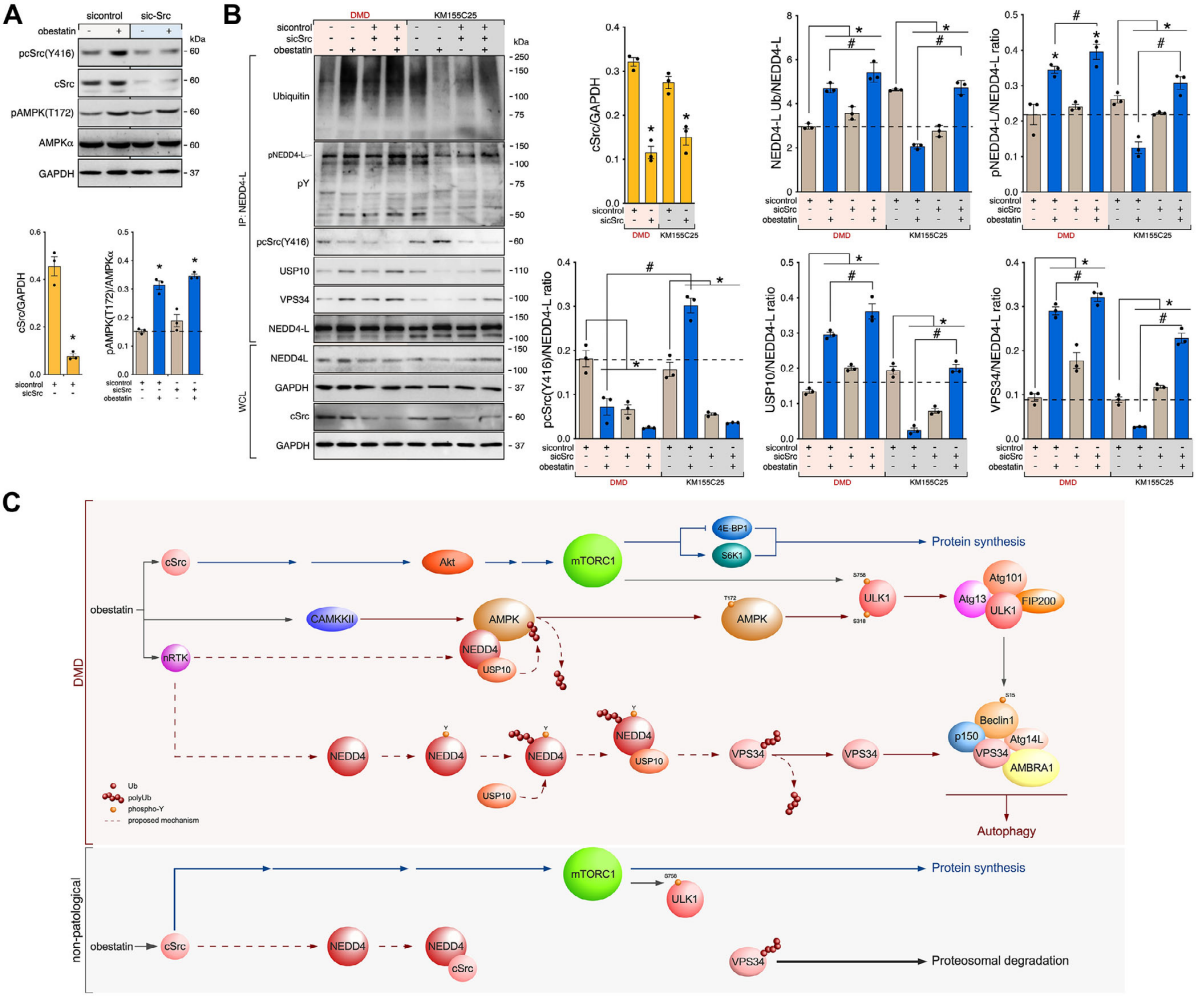
Tyrosine switch on NEDD4‐L facilitates activation of autophagy through the USP10 recruitment and the VPS34 stabilization in human DMD myotubes. (A) Immunoblot analysis of pc‐Src(Y416), c‐Src, pAMPK(T172), or AMPK in DMD myotubes transfected with sicontrol or si‐c‐Src and treated with obestatin (10 nM, 3 h). Data were expressed as the mean ± SEM (*n* = 3 per group; ^*^p < 0.05). (B) Coimmunoprecipitation analysis of DMD cells treated with obestatin (10 nM, 20 min) by using anti‐NEDD4‐L antibody and immunoblotted with ubiquitin, pY, pc‐Src(Y416), USP10, VPS34, or NEDD4‐L antibody. Data were expressed as mean ± SEM (*n* = 3 per group; ^*^p < 0.05). In panels (A) and (B), immunoblots are representative of the mean value. (C) Proposed model by which NEDD4‐L regulates autophagy via obestatin signaling under dystrophic conditions. Tyrosine switch on NEDD4‐L activates autoubiquitination that serves as a scaffold to recruit the deubiquitinase enzyme USP10 to form a deubiquitination complex, which stabilizes VPS34 to promote autophagy through the removal of the ubiquitin chains on VPS34 and activation of Beclin1 complex. In parallel, NEDD4‐L favors AMPK exposure to CaMKKß and consequent activation of ULK1. Under DMD conditions, AMPK does not inhibit mTORC1, but sustains ULK1/Beclin1 activity and autophagy. In nonpathological conditions, lack of action of non‐RTK on NEDD4‐L favors interaction between c‐Src and NEDD4‐L, thus impairing the VPS34 stabilization and AMPK activation.

The family of NEDD4 E3 ubiquitin ligases are related to the regulation of GPCR trafficking [58, 59, 60]. However, little is known about how NEDD4 E3 ligases are released from autoinhibition to increase HECT ubiquitin ligase activity after GPCR stimulation. A recent study showed that c‐Src increases the ubiquitin ligase activity of NEDD4‐L [60]. In this sense, we have previously established that c‐Src acts as a signal node associated to the obestatin/GPR39 system [32]. In fact, obestatin increased c‐Src Y416 phosphorylation, which was abolished by c‐Src‐specific siRNA in DMD cells. Interestingly, T172 phosphorylation of AMPKα was unaffected by depletion of c‐Src in DMD cells (Figure 8A). When we compared DMD and KM155C25 cells after c‐Src knockdown, we found that obestatin significantly increased NEDD4‐L Y phosphorylation in DMD cells and c‐Src deficiency resulted in significant increase in Y phosphorylation in contrast to KM155C25 cells (Figure 8B). When we examined the potential linkage of c‐Src, we found that activated c‐Src was dissociated from NEDD4‐L complex after obestatin treatment in DMD cells, and this dissociation was concomitant to the recruitment of both USP10 and VPS34 (Figure 8B). By contrast, activated c‐Src directly coimmunoprecipitated with NEDD4‐L and this linkage disrupted the USP10–VPS34 binding in KM155C25 cells under obestatin stimulation (Figure 8B). Increased NEDD4‐L ubiquitination coincides with NEDD4‐L Y phosphorylation, dissociation of c‐Src and recruitment of both USP10 and VPS34 in DMD cells in contrast to what was observed in KM155C25 cells (Figure 8B). Thus, obestatin activates autoubiquitination of NEDD4‐L, potentially via NEDD4‐L Y phosphorylation, and functions as a scaffold to recruit USP10 to form a deubiquitination complex. This NEDD4‐L–USP10 complex stabilizes VPS34 by removing the poly‐ubiquitin chains.

## 3 Discussion

Skeletal muscle function requires an effective response to cellular damage, either by repairing it and/or by removing damaged cellular constituents. Extensive research has been conducted to investigate how the accumulation of dysfunctional proteins and defective organelles leads to muscle degeneration [37, 61, 62, 63]. However, the effect that DAPC disassembly has on the autophagic process remains less characterized. In this study, we report the unexpected finding that obestatin functionally integrates mTORC1 and AMPK signaling to control protein synthesis, UPS, and autophagy–lysosome system in a dystrophic context. The pattern of posttranslational modifications of the E3 ligase NEDD4‐L emerges as the major switch to activate the autophagy machinery in response to obestatin, including NEDD4‐L tyrosine phosphorylation and autoubiquitination. NEDD4‐L acts as scaffolding for recruiting USP10 to set up a deubiquitination complex that activates AMPK and stabilizes VPS34 to stimulate autophagy. By reactivating autophagy in dystrophic muscle, obestatin signaling promotes the recovery of physiological skeletal muscle function. This observation is consistent with the decrease in the levels of serum CK, a sign of partial muscle rescue from tissue necrosis. The temporal coupling between mTORC1 and autophagy represents a mechanism in which autolysosome‐derived amino acids reinforce mTORC1 activity, and, in this way, counteract muscle wasting in DMD conditions.

mTORC1 and AMPK are considered as antagonists that suppress or induce autophagy under nutrient sufficiency or shortage, respectively [63, 64, 65, 66, 67, 68, 69, 70]. Our data support a more intricate relationship between AMPK and mTOR. We demonstrate that obestatin signaling attenuates autophagy when energy supplies are sufficient [34, 35], whereas in response to energy stress conditions, such as in DMD, it enhances autophagic flux. Obestatin activates AMPK signaling by a NEDD4‐L‐dependent mechanism to switch autophagy via ULK1 activation while maintaining protein biosynthetic process associated to mTORC1 signaling. The apparent paradox fits with emerging data reveling that proper ULK1 activity requires concurrent AMPK and mTORC1 phosphorylation on distinct sites of ULK1 including S555 (AMPK) [46] and S758 (mTORC1) [46, 71, 72]. Indeed, ULK1 activation elicited by obestatin appears to be positively regulated by mTORC1 activity and mTORC1‐dependent phosphorylation on S758 [46, 71]. This is in agreement with previous data describing the loss of AMPK‐mediated ULK1 phosphorylation upon pharmacological mTORC1 inhibition [73]. Furthermore, concurrent mTORC1 and AMPK activation has been showed to sustain autophagy under amino acid sufficiency [51]. In that case, CaMKKß, but not LKB1, is the AMPK‐activating kinase that drive autophagy initiation as described for obestatin. Intriguingly, CaMKKß was also involved in response to 2‐deoxy‐d‐glucose [74] as well as various other Ca^2+^ mobilizing agents [75]. The fact that AMPK activity was not affected by mTORC1 prompted us to propose a mechanism by which CaMKKß specifically activates an AMPKα isoform whose kinase activity is not inhibited by mTORC1. Indeed, AMPKα1 is primarily activated by CaMKKß [76, 77]. In addition, AMPKα1 is a target for mTORC1, however S356 phosphorylation on AMPKα1 is not related to the inhibition of kinase activity [73]. By contrast, AMPKα2 is directly phosphorylated by mTORC1 at S345, leading to a decrease in kinase activity [78]. S345 phosphorylation reduces the localization of AMPKα2 at the lysosomes, making it unlikely to come into contact with LKB1 [78]. We propose that DMD conditions modify signaling pathways associated with muscle homeostasis and, more importantly, the way in which these modifications affect the functionality of these signals. In particular, dystrophic conditions determine permissiveness to the activation of AMPK complexes containing subunit‐specific isoforms that sustain autophagy under anabolic conditions triggered by obestatin signaling.

As part of an intricate signaling code, ubiquitin encrypts diverse functions that range from enhancing the efficiency of protein degradation to establish large signaling complexes [79]. Our results highlight the role of NEDD4‐L in regulating autophagy, including the regulation of the AMPK activity and VPS34 stabilization under DMD conditions. Regarding AMPK, NEDD4‐L favors the USP10 activation to remove the inhibitory ubiquitination at AMPKα and promote its exposure to CaMKKß. This is supported by a study that demonstrates deubiquitination and activation of AMPK by USP10 in response to fluctuations of cellular energy status [80]. In relation to VPS34, the assembly of the NEDD4‐L–USP10 complex is critical for cleaving the poly‐ubiquitin chains at VPS34, thereby promoting the formation of a functional VPS34 complex. This is not an exceptional case and, in fact, it has been reported that several E3 ubiquitin ligases and deubiquitinating enzymes target the VPS34 complex [56, 81, 82, 83]. Beyond the molecular mechanism for autophagy cascade, an important piece of this puzzle is the mode by which NEDD4‐L is released from autoinhibition following obestatin stimulation. Intramolecular interactions normally prevent the autoubiquitylation of NEDD4 E3 ligases, but this inhibition can be overcome by phosphorylation or binding of accessory proteins [84]. However, the mode of regulation of NEDD4‐L differs between DMD and nonpathological muscle. The autoubiquitination of NEDD4‐L and the recruitment of USP10 appear to be associated with the tyrosine phosphorylation of NEDD4‐L. Interestingly, c‐Src‐dependent tyrosine phosphorylation of NEDD4 was shown to promote its ubiquitin ligase activity [56, 85]. However, c‐Src is disengaged from the NEDD4‐L after obestatin treatment under dystrophic conditions, and strikingly, depletion of c‐Src promotes the tyrosine phosphorylation of NEDD4‐L and assembly of NEDD4‐L–USP10–VPS34 complex, indicating a negative regulation on this complex. Notably, the NEDD4‐L–USP10–VPS34 signaling complex was absent in nonpathological muscle cells in which obestatin‐activated c‐Src is associated to NEDD4‐L and its tyrosine phosphorylation was deficient. Nonetheless, these data emphasize the physiological function for the tyrosine switch in NEDD4‐L activation and the obestatin‐induced autophagy signaling in a dystrophic context. Further studies are required to determine the specific role of nonreceptor protein tyrosine kinase‐mediated NEDD4‐L activation in obestatin‐induced autophagy.

Autophagy is a key process in the regulation of muscle mass, integrity and function [23, 86]. Administration of obestatin rescued autophagy in *mdx* mice and myotubes from DMD patients. The positive impact on autophagy was reflected in improved muscle function in *mdx* mouse model of DMD. The conversion of fast‐to‐slow twitch fibers, which are more resistant to damage, may be partly responsible for this gain of function [33]. This observation is consistent with previous findings on *mdx* mice, in which obestatin was shown to act by both HDAC/Mef2 and PGC1α mechanisms [36]. In addition, obestatin treatment shifted the balance from protein degradation to protein synthesis. This is supported by the downregulation of the E3 ubiquitin ligases MAFbx and MuRF1, hallmarks of skeletal muscle atrophy, and activation of key nodes in protein synthesis, S6K1 and eIF4E. The effect of obestatin on both the removal and recycling of dysfunctional proteins and organelles is in line with the coordinated activation of AMPK and mTOR pathways. This action ensures the efficient deployment of cellular resources in DMD conditions. Indeed, the analyses of MHC expression indicates that blockage of autophagy was sufficient to block protein biosynthetic process associated to obestatin signaling in DMD conditions. This is even more relevant given the contribution of preproghrelin, and thus obestatin, to proper autophagic function during myogenic differentiation. Depletion of preproghrelin was sufficient to inhibit the conversion of LC3I to LC3II and protein sets related to autophagy in human myoblasts. The decrease in the expression of the cell proliferation marker Ki‐67 and the myogenic regulatory factor myogenin further indicates a myogenic impairment. In fact, the absence of MHC protein in preproghrelin silenced cells supports a role in human myogenesis. At this point, it is reasonable to speculate about the significance of obestatin as a limiting factor not only for the activation of the myogenic program but also for proper autophagy in DMD conditions. This hypothesis is supported by the ageing‐related preproghrelin expression, correlating with impaired autophagic activity in DMD. This fact is even more meaningful considering that preproghrelin expression is linked to mitochondrial dynamic markers critical to maintain cell regenerative competence [87, 88]. In particular, preproghrelin loss in human myoblasts impairs the mitochondrial fission regulator DRP1, the mitophagy regulator PARKIN1, and the core mitochondrial transcription factor TFAM. These findings point to a model in which mitochondria dynamics is somehow associated to preproghrelin expression by determining mtDNA transcription and replication, as well as mitochondrial segregation and elimination, which are critical functions in the switch from quiescence to the proliferative fate of these cells during tissue repair [88, 89, 90, 91, 92, 93, 94]. Therefore, it is certainly tempting to speculate that the age‐related loss of preproghrelin contributes to the reduction in autophagy activity in DMD. This leads to the accumulation of damaged cellular components, including mitochondria, which may contribute to the impaired ability to regenerate muscle tissue.

Defects in lysosomal function [95, 96] or in the autophagy pathway [97, 98, 99] are directly associated to muscular dystrophy. Of note, obestatin treatment of *mdx* mice not only restores autophagy flux, but also unblocks lysosome fusion to autophagic vesicles. Indeed, obestatin signaling reverses the marked accumulation of p62 with enhanced colocalization between LAMP2 and p62 in *mdx* mice. Our study not only confirms the importance of obestatin in the autophagic process, but also indicates that is required for proper autophagic function in dystrophic muscle conditions. These findings are in accordance with substantial improvement in the skeletal function and integrity after obestatin treatment in *mdx* mice. Signs of attenuation of proteotoxicity by increasing basal autophagy flux, correlating with decreased ROS levels and effective myogenic functions, were also observed in human DMD cells.

The absence of dystrophin deteriorates the sarcolemma, rendering it vulnerable to contraction‐induced damage. Over time, all these alterations are transferred to the nucleus and abnormal mechanotransduction encourages abnormal gene expression [100, 101]. Indeed, the loss of dystrophin disrupts preproghrelin expression and alters obestatin signaling, which contributes to muscle dysfunction in this disease. Our study denotes that NEDD4‐L serves as a gatekeeper between anti‐ and proautophagic response associated to the obestatin/GPR39 system in DMD conditions, which is essential for the decision‐making process during autophagy. Obestatin led to activation of a nonreceptor tyrosine kinase, which subsequently tyrosine‐phosphorylate NEDD4‐L and promotes autoubiquitination as a platform for recruiting deubiquitinating enzyme USP10 to cleave the ubiquitination of VPS34. The NEDD4‐L–USP10 axis suppresses the proteasomal degradation of VPS34 and promotes NEDD4‐L–USP10–VPS34 signaling complex via non receptor tyrosine kinase‐dependent activity on NEDD4‐L. In addition, activation of NEDD4‐L favors AMPK exposure to CaMKKß through USP10 (Figure 8C). The activation of the CaMKKß–AMPK axis by obestatin does not inhibit mTORC1 signaling, but sustains ULK1 activity and autophagy, contrary to what happens in normal muscle. Restoration of constitutive autophagy prevents intracellular damage accumulation, and counteracts functional decline of dystrophic muscle, offering unexpecting therapeutic targets in dystrophic muscle. How muscle preproghrelin synthesis is regulated, and how dystrophin and dystrophin–glycoprotein complex participate to this regulation, still need to be explored. Our findings reveal the existence of additional signaling targets in the dystrophic context, and validate future research to evaluate whether obestatin can provide therapeutic benefit to dystrophic patients through the partial recovery of muscle homeostasis.

The models used in this study have some limitations. The *mdx* mouse is the model of choice in DMD research by the convenience and cost‐effectiveness. However, this convenience is counterbalanced by the fact that *mdx* myopathy does not accurately resemble human pathology. To be as close as feasible to the human disorder, immortalized human DMD myoblasts were used. Although the two DMD models and their controls were used, it remains uncertain whether these phenotypes represent concurrent phenotypes of all type of mutations in DMD patients. To the above, we must add the absence of the complex microenvironment of surrounding myofibers. This dynamic environment enables muscle repair and regeneration. Therefore, further studies are necessary to achieve the validation from our study, and to determine its clinical significance.

This study reveals that obestatin integrates the mTORC1 and AMPK signaling pathways in DMD conditions. This action reactivates protein synthesis and the ubiquitin–proteasome and autophagy–lysosome systems. The restoration of skeletal muscle homeostasis is the overall effect of obestatin signaling in dystrophic conditions.

## 4 Materials and Methods

### 4.1 Materials

Mouse and human obestatin were obtained from BCN Peptides (Barcelona, Spain). Table S1 shows the antibodies that were used in this work. Sigma Chemical Co (St. Louis, MO, USA) supplied all other chemical reagents.

### 4.2 Animals and Obestatin Dosing

This study used 4‐, 8‐, and 18‐week‐old male C57BL/10ScSn–Dmdmdx/J mice (*mdx* mice) obtained from The Jackson Laboratory (Bar Harbor, ME, USA). For protein expression assays, 4‐, 8‐, and 18‐week‐old male *mdx* mice were used (*n* = 3 per age group). For obestatin dosing assays, 8‐week‐old male *mdx* mice were divided to two experimental groups: (1) control group (vehicle: PBS [pH 6.3]; *n* = 5); and, (2) obestatin‐treated group (*n* = 5). Vehicle or obestatin solution in PBS (500 nmol/kg body weight) was injected intramuscularly into TA muscle every 72 h during 30 days [33, 36]. The mice were sacrificed 30 days postinjection and TA muscles were harvested, and processed for subsequent analyses. Animal experiments were carried out in accordance with the European Community Directive 2010/63/EU and Spanish law (Real Decreto 53/2013). The experimental procedures were approved by the University of Santiago de Compostela Animal Care Committee (approval no. 15010/17/005).

### 4.3 Measurements of Muscle Force in Vivo

Muscle force was measured as previously described [36]. Force was measured in vivo in anaesthetized mice using 1305A Whole Animal System (Aurora Scientific, Inc., ON, Canada).

### 4.4 Blood Measurements

Blood samples were obtained from tail bleeds under general anesthesia at the end of the procedure. CK analysis was performed at the Central Laboratory at Hospital Clínico Universitario de Santiago de Compostela (Santiago de Compostela, Spain).

### 4.5 Cell Culture and Differentiation of Human Immortalized DMD Myoblasts

DMD6 and DMD14 myogenic clonal cell lines were obtained from the Centre for Research in Myology (Paris, FR). The isolation and immortalization were performed from biopsies obtained through MYOBANK (EU network EuroBioBank), in accordance with French legislation and European recommendations [36, 101, 102, 103, 104]. In particular, deltoid and paravertebral muscle biopsies were obtained from 6‐ and 14‐year‐old male patients suffering from DMD (duplication of exon 2), respectively. Myoblasts were cultivated in growth medium (GM) containing Medium 199:DMEM (1:4, v:v; Lonza, Pontevedra, Spain) supplemented with 20% FBS (v/v), 25 µg/µL fetuin, 5 ng/mL hEGF, 0.5 ng/mL bFGF, 0.2 µg/mL Dexa, and 50 µg/mL gentamycin (Invitrogen, ThermoFisher Scientific; MA, USA). Myotube differentiation was initiated at 90% confluence by switching to differentiation medium (DM; DMEM supplemented 50 µg/mL gentamycin [Invitrogen]) for 3 days.

### 4.6 Cell Culture and Differentiation of human Immortalized Myoblasts

Myogenic clonal line, KM155C25 Clone 48 (KM155C25 cells), was obtained from the Centre for Research in Myology (Paris, FR). The isolation and immortalization were developed from gracilis muscle biopsy (donor age 25 years) obtained through MYOBANK (EU network EuroBioBank) following the protocols previously described [34, 100]. KM155C25 cells were cultivated in GM. Differentiation into myotubes was initiated at 90% confluence by switching to DM for 4 days unless otherwise stated.

### 4.7 In Vitro Treatments

Human DMD or KM155C25 cells were treated with obestatin (10 nM), insulin (1.72 µM), or Dexa (1 µM) in GM or DM for myoblast or myotubes, respectively. Chloroquine (20 µM) was used to block autophagy for 6 h at 37°C. Bafilomycin A1 (100 nM) was used to inhibit the acidification of lysosomes for 4 h at 37°C. Compound C (10 µM) was used to inhibit AMPK for 30 min at 37°C. STO‐609 (25 µM) was used to inhibit CaMKKβ for 1 h at 37°C. MG132 (100 µM) was used to block the proteolytic activity of the 26S proteasome complex for 6 h at 37°C unless otherwise stated.

### 4.8 Histology and Immunofluorescence Analysis

For tissue analysis, excised muscles were embedded in tragacanth gum, and snap‐frozen in liquid nitrogen‐cooled isopentane. Cryosections at 8‐µm thickness were mounted on Histobond Adhesion Microslides (Marienfeld, Lauda‐Königshofen, Germany). HE and SDH staining were developed by using standard procedures. Immunofluorescence analysis was conducted using protocols previously described [35, 36]. For myogenic cell analysis, the myoblasts were cultured on coverslips and differentiated into myotubes. Cells were processed according to protocols previously described [35, 36]. 4′,6‐Diamidino‐2‐phenylindole (DAPI) was used to counterstain the cell nuclei (Invitrogen). Images were collected blind (>3 images per condition) from three independent repeats. Mean fluorescence intensity (MFI) and Pearson's correlation coefficients (*r*) were calculated using ImageJ software. The digital images were taken with a Leica TCS‐SP8 spectral confocal microscope (Leica Microsystems, Heidelberg, Germany).

### 4.9 Immunoblot Analysis

Protein was isolated from cells or tissue using standard methods previously described [35, 36]. Immunoreactive bands were detected by enhanced chemiluminescence (Pierce ECL Western Blotting Substrate; Thermo Fisher Scientific, Rockford, IL, USA). Band intensities were analyzed by NIH Image software, ImageJ 1.5b (National Institutes of Health, Bethesda, MD, USA).

### 4.10 ROS Detection

CellROX Green Reagent (Invitrogen) was used following the manufacturer's instructions and directly analyzed after fixation using fluorescence microscopy.

### 4.11 siRNA‐ and Short Hairpin RNA‐Mediated Gene Silencing

To knockdown NEDD4, AMPKα or USP10 expression, siRNA specifically targeting human NEDD4‐L (sc‐75894; Santa Cruz Biotechnology, CA, USA), AMPKα (sc‐45312; Santa Cruz Biotechnology), or USP10 (sc‐76811; Santa Cruz Biotechnology) was used. siRNA duplexes targeting preproghrelin or c‐Src were selected from ON‐TARGET plus SMARTpool siRNA from Thermo Fisher Scientific (Dharmacon, CO, USA). shRNA‐mediated gene silencing was used for ULK1 knockdown (sc‐44182‐SH; Santa Cruz Biotechnology) in DMD cells. An ON‐TARGETplus nontargeting siRNA (Dharmacon) or control nontargeting shRNA plasmid (sc‐108060; Santa Cruz Biotechnology) was used as a control for siRNA or shRNA experiments. Cells were transfected with Lipofectamine 2000 (Invitrogen) following the manufacturer's instructions.

### 4.12 Plasmids and Transfection

ptfLC3 (Addgene plasmid # 21074; http://n2t.net/addgene:21074; RRID: Addgene 21074) was a gift from Prof. Tamotsu Yoshimori [105]. pCI HA NEDD4L (Addgene plasmid # 27000; http://n2t.net/addgene:27000; RRID: Addgene 27000) and pCI HA NEDD4L DD (Addgene plasmid # 27001; http://n2t.net/addgene:27001; RRID: Addgene 27001) were a gift from Prof. Joan Massague [106]. pFRT_TO_FlagHA_USP10 (Addgene plasmid # 127102; http://n2t.net/addgene:127102; RRID: Addgene 127102) and pFRT_TO_FlagHA_USP10_C424A (Addgene plasmid # 127103; http://n2t.net/addgene:127103; RRID: Addgene_127103) were a gift from Prof. Thomas Tuschl [107]. Cell transfections were performed using linear polyethylenimine (MW 25,000; Kyfora Bio, PA, USA) according to procedures recommended by the manufacturer.

### 4.13 Coimmunoprecipitation Assays

Following treatment, DMD or KM155C25 myotube cells were washed twice with ice‐cold PBS and lysed in coimmunoprecipitation lysis buffer (50 mM Tris, 100 mM NaCl, 5 mM EDTA, 50 mM NaF, 1% Triton X‐100 (v/v), 10 mM glycerol phosphate, 200 µM sodium orthovanadate, 2.5 mM sodium pyrophosphate plus protease inhibitors). The primary antibodies were coupled to Protein A Magnetic Beads according to instruction provided by manufacturer (Cell Signalling Technology, MA, USA). The washed immunoprecipitates were subjected to immunoblot analysis using the indicated antibodies (Table S1).

### 4.14 Mitochondrial analysis

Human DMD myoblast cells were differentiated on coverslips. Live myotubes were loaded with MTDR (Invitrogen) after obestatin (10 nM) or vehicle treatment (24 h) following manufacturer's instructions.

### 4.15 Statistics

Values were presented as mean ± standard error of the mean (SEM). A Shapiro–Wilks normality test was performed for each data set. Comparisons between two samples were carried out using *T*‐test. Unpaired *t*‐test was used to assess the statistical significance of one‐way or two‐way analysis when the test statistic followed a normal distribution. *p* < 0.05 was considered as statistically significant.

## Author Contributions

Conceptualization: Y.P. and J.P.C. Formal analysis: I.S.Z., S.C.A., A.C.L., F.F.B., T.C.D., S.L.L., V.M., X.C., R.G., and J.L.R. Investigation: I.S.Z., S.C.A., A.C.L., F.F.B., T.C.D., S.L.L., J.G.S., M.G.L., L.D.C., K.M., and C.S.M. Data analysis: I.S.Z., S.C.A., A.C.L., T.C.D., S.L.L., Y.P.R., and J.P.C. Writing: I.S.Z., V.M., X.C., Y.P.R., and J.P.C. Funding acquisitions: I.S.Z., Y.P.R., and J.P.C. All authors have read and approved the final manuscript.

## Funding

This work was supported by grants from Instituto de Salud Carlos III in cofinancing with FEDER (ISCIII‐FEDER; MINECO, Spain; PI18/00760, PI21/01639, PI22/00155, and PI24/01602), “la Caixa” Foundation (ID 100010434) and Duchenne Parent Project España. The work of Icía Santos‐Zas is funded by “la Caixa” Foundation (LCF/BQ/PI22/11910038). IDIS funds Silvia Costas‐Abalde through a predoctorate research scholarship. Xunta de Galicia funds Andrea Calviño‐Lodeiro through a predoctorate research scholarship, Axencia Galega de Innovacion, Xunta de Galicia (IN607D2023/04, IN607D2024/05).

## Conflicts of Interest

The authors declare no conflicts of interest.

## Ethics Statement

The protocols for animal experiments were approved by the Bioethics Committee at the University of Santiago de Compostela according to the guidelines of the Spanish Royal Decree 53/2013, Directive 2010/63/EU, and FELASA Guidelines (approval no. 15010/17/005).

## Supporting information




**Supporting Table 1**: Primary antibodies. Relation of the primary antibodies used in the different analyses performed in this work. WB, western blot; IF, immunofluorescence; IP, coimmunoprecipitation.
**Supporting Figure 1**: (A) DMD myotubes were pretreated with Compound C previous to obestatin stimulation (10 nM, 30 min). pULK1(S318), pULK1(S758), pULK1(S555), pAMPK(T172), pmTOR(S2448), p62, and LC3 were analyzed by immunoblot. Data were expressed as mean ± SEM obtained from intensity scans (*n* = 3; ^*^p < 0.05). (B) DMD myotubes were pretreated with STO‐609 previous to obestatin stimulation (10 nM, 30 min). pULK1(S555), pAMPK(T172), p62, and LC3 were analyzed by immunoblot. Data were expressed as mean ± SEM (*n* = 3; ^*^ p < 0.05). (C) DMD myotubes were transfected with shRNA targeting human ULK1 or shRNA‐scramble prior to stimulation with obestatin (10 nM, 30 min) in the presence or absence of chloroquine treatment. The expression of ULK1, p62, and LC3 was evaluated by immunoblot analysis. Data were expressed as the mean ± SEM (*n* = 3; ^*^p < 0.05). (D) Protein extract from obestatin‐treated DMD or KM155C25 myotubes (10 nM) were subjected to immunoprecipitation using anti‐ULK1 antibody. Immunopurified complexes were analyzed by immunoblot to detect PP1, PP2A, PP5, αOGT, αO‐GlcNAc, ULK1‐linked ubiquitin [(Ub)_n_‐ULK1], AMBRA1, TRIM32, and mTOR. These proteins were also analyzed in total extracts (*n* = 3). To ensure a fair comparison, the amounts of immunoprecipitates loaded for Western blot were adjusted to allow an equal level of VPS34 appearing in DMD and KM155C25 cells. In panels (A)–(D), the immunoblots are representative of the mean value.
**Supporting Figure 2**: (A) Representative images of MitoTracker Deep Red (MTDR) staining of human DMD myotubes exposed to vehicle (PBS) or obestatin (10 nM) to assess mitochondrial analysis. Right panel, the changes in mean fluorescence intensity (MFI) of MTDR is shown. Data were expressed as arbitrary units (au; *n* = 5 per group; mean ± SEM; ^*^p < 0.05). (B) Immunoblot analysis of PGC1α and TOM20 in the TAs from mdx mice after intramuscular injection of obestatin (500 nmol/kg body weight each 72 h; *n* = 5) or vehicle (control; *n* = 5) during 30 days. Data were expressed as the mean ± SEM obtained from intensity scans (^*^p < 0.05). (C) Representative images of vehicle‐ and obestatin‐treated TAs showing TOM20 expression. The changes in MFI of TOM20 are shown (*n* = 5 per group). Data were expressed as mean ± SEM as au (^*^p < 0.05).
**Supporting Figure 3**: (A) Schematic of mRFP–EGFP–LC3 reporter. When the reporter is associated to autophagosomes, both mRFP and EGFP fluoresce and autophagosomes are visualized as yellow puncta. Once the autophagosome fuses with a lysosome GFP fluorescence is quenched and autophagolysosomes appear as red fluorescent puncta. (B) Autophagy activity in DMD and KM155C25 myoblast cells expressing mRFP–GFP–LC3 treated with obestatin (10 nM) or PBS (control) for 24 h in the presence or absence of bafilomycin treatment. (C) Quantification of autophagosome vesicles. (D) Quantification of autolysosome vesicles. In C and D, insets show higher magnification and nuclei are highlighted with DAPI. Values are mean ± SEM (*n* = 50 cells from 6 independent experiments; ^*^p < 0.05).

## Data Availability

All data are available from the corresponding authors upon reasonable request.
