# Supplementary material for: Obestatin Treatment Counteracts Muscle Wasting by Reactivation of Autophagy in Duchenne Muscular Dystrophy
Source: MedComm (2020). 2026 Jan 14;7(1):e70563. doi: 10.1002/mco2.70563 (PMC12803508; doi:10.1002/mco2.70563)
Supplement: Supplementary file 1 — Supporting Table 1: Primary antibodies. Relation of the primary antibodies used in the different analyses performed in this work. WB, western blot; IF, immunofluorescence; IP, coimmunoprecipitation. Supporting Figure 1: (A) DMD myotubes were pretreated with Compound C previous to obestatin stimulation (10 nM, 30 min). pULK1(S318), pULK1(S758), pULK1(S555), pAMPK(T172), pmTOR(S2448), p62, and LC3 were analyzed by immunoblot. Data were expressed as mean ± SEM obtained from intensity scans (n = 3; *p < 0.05). (B) DMD myotubes were pretreated with STO‐609 previous to obestatin stimulation (10 nM, 30 min). pULK1(S555), pAMPK(T172), p62, and LC3 were analyzed by immunoblot. Data were expressed as mean ± SEM (n = 3; * p < 0.05). (C) DMD myotubes were transfected with shRNA targeting human ULK1 or shRNA‐scramble prior to stimulation with obestatin (10 nM, 30 min) in the presence or absence of chloroquine treatment. The expression of ULK1, p62, and LC3 was evaluated by immunoblot analysis. Data were expressed as the mean ± SEM (n = 3; *p < 0.05). (D) Protein extract from obestatin‐treated DMD or KM155C25 myotubes (10 nM) were subjected to immunoprecipitation using anti‐ULK1 antibody. Immunopurified complexes were analyzed by immunoblot to detect PP1, PP2A, PP5, αOGT, αO‐GlcNAc, ULK1‐linked ubiquitin [(Ub)n‐ULK1], AMBRA1, TRIM32, and mTOR. These proteins were also analyzed in total extracts (n = 3). To ensure a fair comparison, the amounts of immunoprecipitates loaded for Western blot were adjusted to allow an equal level of VPS34 appearing in DMD and KM155C25 cells. In panels (A)–(D), the immunoblots are representative of the mean value. Supporting Figure 2: (A) Representative images of MitoTracker Deep Red (MTDR) staining of human DMD myotubes exposed to vehicle (PBS) or obestatin (10 nM) to assess mitochondrial analysis. Right panel, the changes in mean fluorescence intensity (MFI) of MTDR is shown. Data were expressed as arbitrary units (au; n = 5 per group; mean ± SEM; * [file MCO2-7-e70563-s001.docx]

**Obestatin treatment counteracts muscle wasting by reactivation of autophagy in Duchenne muscular dystrophy**

**Running title:** Obestatin treatment reactivates autophagy in DMD

Icía Santos-Zas^1#^, Silvia Costas-Abalde^1,2#^, Andrea C. Lodeiro^1,2^, Fátima Fernández-Barreiro^1^, Tania Cid-Díaz^1^, Saúl Leal-López^2^, Jessica González-Sánchez^1^, Mar García-Lamela^1^, Lucía Debasa-Corral^1^, Carlos S. Mosteiro^1^, Kamel Mamchaoui^3^, Vincent Mouly^3^, Xesús Casabiell^1,4^, Rosalía Gallego^5^, José Luis Relova^1,4^, Yolanda Pazos^2,*^, Jesus P. Camiña^1,**^

(1) Grupo de Endocrinología Celular, Instituto de Investigación Sanitaria de Santiago (IDIS), Complejo Hospitalario Universitario de Santiago (CHUS), Servicio Gallego de Salud (SERGAS), Trav. Choupana s/n, Santiago de Compostela, Spain.

(2) Grupo de Investigación Traslacional en Enfermedades del Aparato Digestivo (GITEAD), IDIS, CHUS, SERGAS, Trav. Choupana s/n, Santiago de Compostela, Spain.

(3) Sorbonne Université, Inserm, Institut de Myologie, Centre de Recherche en Myologie, F-75013 Paris, France.

(4) Departamento de Fisiología, Universidade de Santiago de Compostela (USC), Santiago de Compostela, Spain.

(5) Departamento de Ciencias Morfológicas, USC, Santiago de Compostela, Spain.

# These authors contributed equally to this work

*Corresponding author. Tel: +34 981 955075; E-mail: [yolanda.pazos.randulfe@sergas.es](mailto:yolanda.pazos.randulfe@sergas.es)

**Corresponding author. Tel: +34 981 955072; E-mail: [jesus.perez@usc.es](mailto:jesus.perez@usc.es) and jesus.perez.camina@sergas.es

**Table S1. Primary antibodies.** Relation of the primary antibodies used in the different analyses performed in this work. WB, western blot; IF, immunofluorescence; IP, co-immunoprecipitation.

| **Primary antibody** | **Supplier** | **Reference** | **Use** |
| --- | --- | --- | --- |
| 4EBP1 | Cell Signaling | 9452 | WB |
| p4EBP1 (T37/46) | Cell Signaling | 2855 | WB |
| AMBRA1 | Santa Cruz | sc-398204 | WB |
| AMPKα | Cell Signaling | 2532 | WB,IP |
| pAMPK (Thr172) | Cell Signaling | 2535 | WB |
| Atg14 | Cell Signaling | 96752 | WB |
| Beclin1 | Cell Signaling | 3495 | WB |
| pBeclin1 (S15) | Invitrogen | PA5-104576 | WB |
| Bnip3 | Santa Cruz | sc-56167 | WB |
| Cathepsin L | Santa Cruz | sc-390385 | WB |
| DRP1 | Cell Signaling | 8570 | WB |
| GAPDH | Santa Cruz | sc-32233 | WB |
| GPR39 | Novus Biologicals | NLS139 | WB |
| HA | Sigma | H9658 | WB |
| Ki-67 | Santa Cruz | sc-23900 | WB |
| K48-linkage specific polyubiquitin | Cell Signaling | 8081 | WB |
| K63-linkage specific polyubiquitin | Cell Signaling | 5621 | WB |
| LAMP2 | Santa Cruz | sc-18822 | IF |
| LC3A/B | Cell Signaling | 12741 | WB |
| LC3B | Cell Signaling | 2775 | IF |
| MHC | DSHB | MF 20 | IF |
| MAFbx | Santa Cruz | sc-166806 | WB |
| Murf1 | Santa Cruz | sc-398608 | WB |
| mTOR | Cell Signaling | 2972 | WB |
| pmTOR (S2448) | Cell Signaling | 5536 | WB |
| Mfn2 | Santa Cruz | sc-515647 | WB |
| Myogenin | Santa Cruz | sc-576 | WB |
| NEDD4-L |  | 4013 | WB,IP |
| p21 | Cell Signaling | 37543 | WB |
| p62 | Cell Signaling | 5114 | WB |
| p62 | Cell Signaling | 5114 | IF |
| Parkin | Santa Cruz | sc-133167 | WB |
| PGC1α | Invitrogen | PA5-72948 | WB |
| Pink1 | Santa Cruz | sc-8056 | WB |
| PP1 | Santa Cruz | sc-7482 | WB |
| PP2A-Aα | Santa Cruz | sc-56954 | WB |
| PP5 | Santa Cruz | sc-271816 | WB |
| preproghrelin | Santa Cruz | sc-10368 | WB |
| S6 | Cell Signaling | 2217 | WB |
| pS6 (S235/236) | Cell Signaling | 2211 | WB |
| pS6 (S240/244) | Cell Signaling | 2215 | WB |
| c-Src | Santa Cruz | sc-8056 | WB |
| pc-Src(Y416) | Cell Signaling | 2101 | WB |
| TOM20 | Santa Cruz | sc-17764 | WB,IF |
| TFAM | Cell Signaling | 8076 | WB |
| TRIM32 | Santa Cruz | sc-135588 | WB |
| Ubiquitin | Cell Signaling | 3933 | WB |
| ULK1 | Cell Signaling | 8054 | WB |
| pULK1 (S317) | Abgent | AP19250c | WB |
| pULK1 (S758) | GeneTex | GTX132654 | WB |
| pULK1 (S555) | Cell Signaling | 5869 | WB |
| p-Y | Cell Signaling | 9411 | WB |
| UVRAG | Cell Signaling | 13115 | WB |
| USP10 | Cell Signaling | 5553 | WB,IP |
| USP13 | Cell Signaling | 12577 | WB |
| VPS34 | Cell Signaling | 4263 | WB,IP |
| α-OGT | Santa Cruz | sc-74546 | WB |
| αO-GlcNac | Santa Cruz | sc-59623 | WB |

**
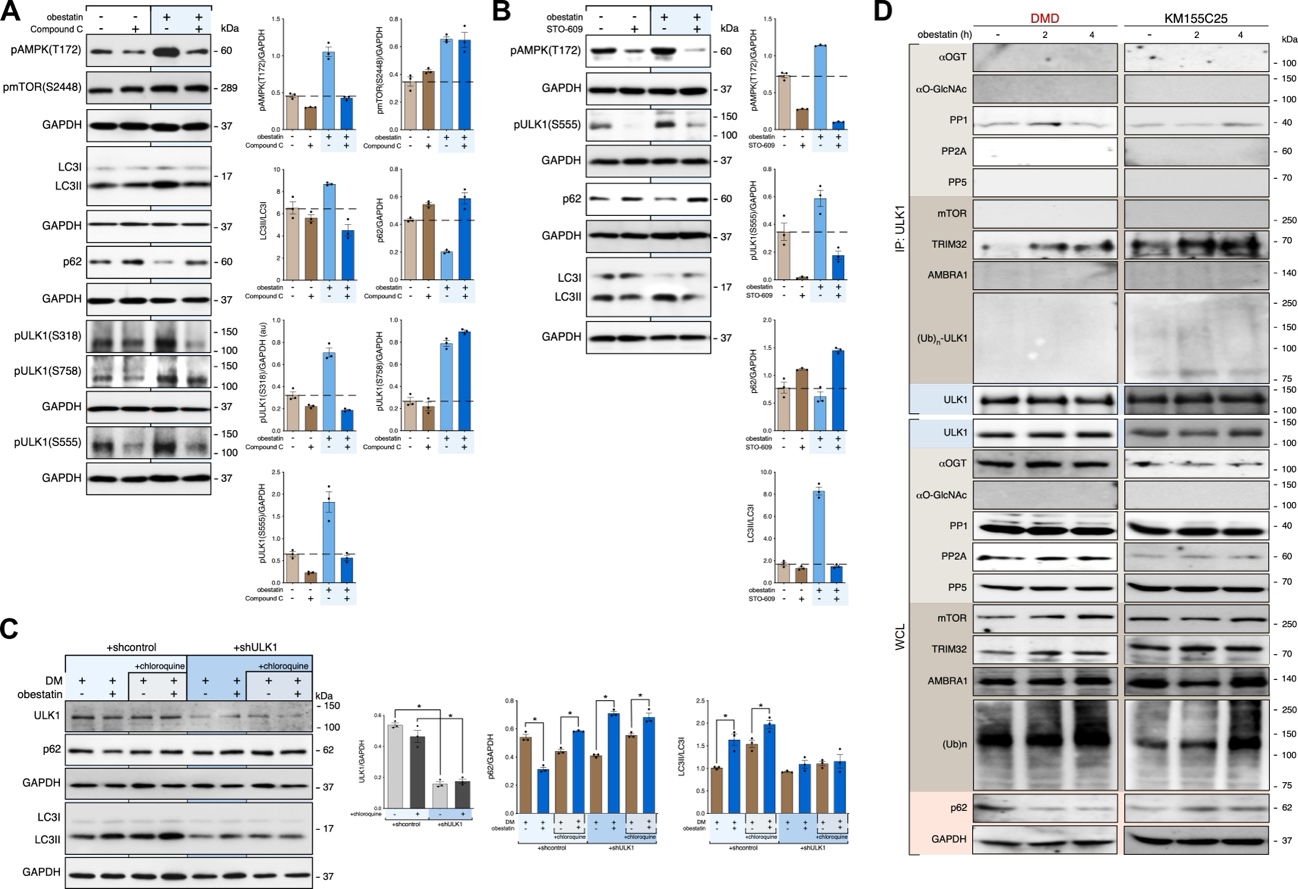
**

**FIGURE S1** (**A**) DMD myotubes were pretreated with Compound C previous to obestatin stimulation (10 nM, 30 min). pULK1(S318), pULK1(S758), pULK1(S555), pAMPK(T172), pmTOR(S2448), p62, and LC3 were analysed by immunoblot. Data were expressed as mean ± SEM obtained from intensity scans (n = 3; *^*^* *p* < 0.05). (**B**) DMD myotubes were pretreated with STO-609 previous to obestatin stimulation (10 nM, 30 min). pULK1(S555), pAMPK(T172), p62, and LC3 were analysed by immunoblot. Data were expressed as mean ± SEM (n = 3; *^*^* *p* < 0.05). (**C**) DMD myotubes were transfected with shRNA targeting human ULK1 or shRNA-scramble prior to stimulation with obestatin (10 nM, 30 min) in the presence or absence of chloroquine treatment. The expression of ULK1, p62, and LC3 was evaluated by immunoblot analysis. Data were expressed as the mean ± SEM (n = 3; *^*^* *p* < 0.05). (**D**) Protein extract from obestatin-treated DMD or KM155C25 myotubes (10 nM) were subjected to immunoprecipitation using anti-ULK1 antibody. Immunopurified complexes were analysed by immunoblot to detect PP1, PP2A, PP5, αOGT, αO-GlcNAc, ULK1-linked ubiquitin [(Ub)_n_-ULK1], AMBRA1, TRIM32, and mTOR. These proteins were also analysed in total extracts (n = 3). To ensure a fair comparison, the amounts of immunoprecipitates loaded for Western blot were adjusted to allow an equal level of VPS34 appearing in DMD and KM155C25 cells. In A-D, the immunoblots are representative of the mean value.

**
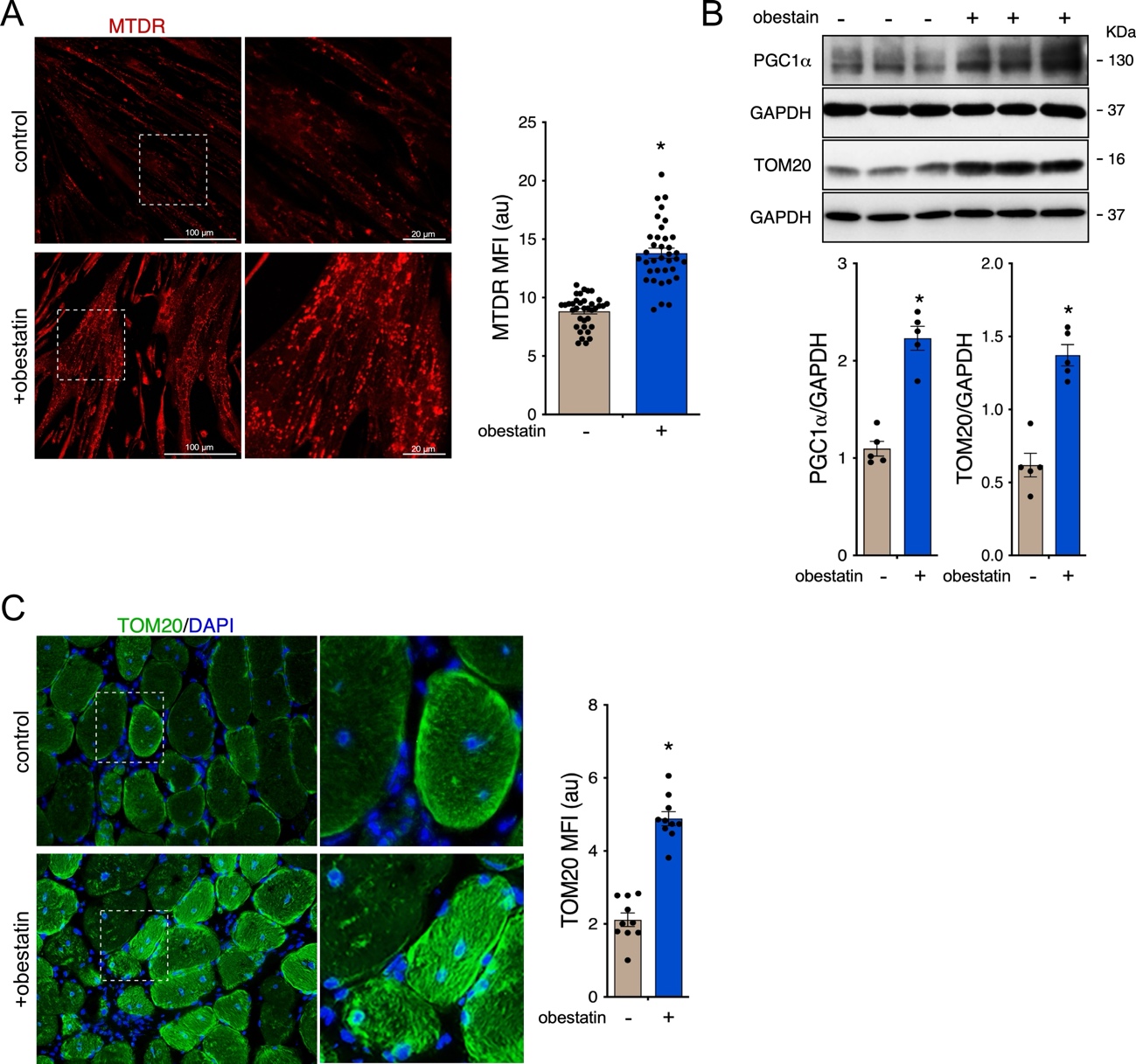
**

**FIGURE S2** (**A**) Representative images of MitoTracker Deep Red^®^ (MTDR) staining of human DMD myotubes exposed to vehicle (PBS) or obestatin (10 nM) to assess mitochondrial analysis. Right panel, the changes in mean fluorescence intensity (MFI) of MTDR is shown. Data were expressed as arbitrary units (au; n = 5 per group; mean ± SEM; *^*^* *p* < 0.05). (**B**) Immunoblot analysis of PGC1α and TOM20 in the TAs from mdx mice after intramuscular injection of obestatin (500 nmol/kg body weight each 72 h; n = 5) or vehicle (control; n = 5) during 30 days. Data were expressed as the mean ± SEM obtained from intensity scans (*^*^* *p* < 0.05). (**C**) Representative images of vehicle- and obestatin-treated TAs showing TOM20 expression. The changes in MFI of TOM20 are shown (n = 5 per group). Data were expressed as mean ± SEM as au (*^*^* *p* < 0.05).

**
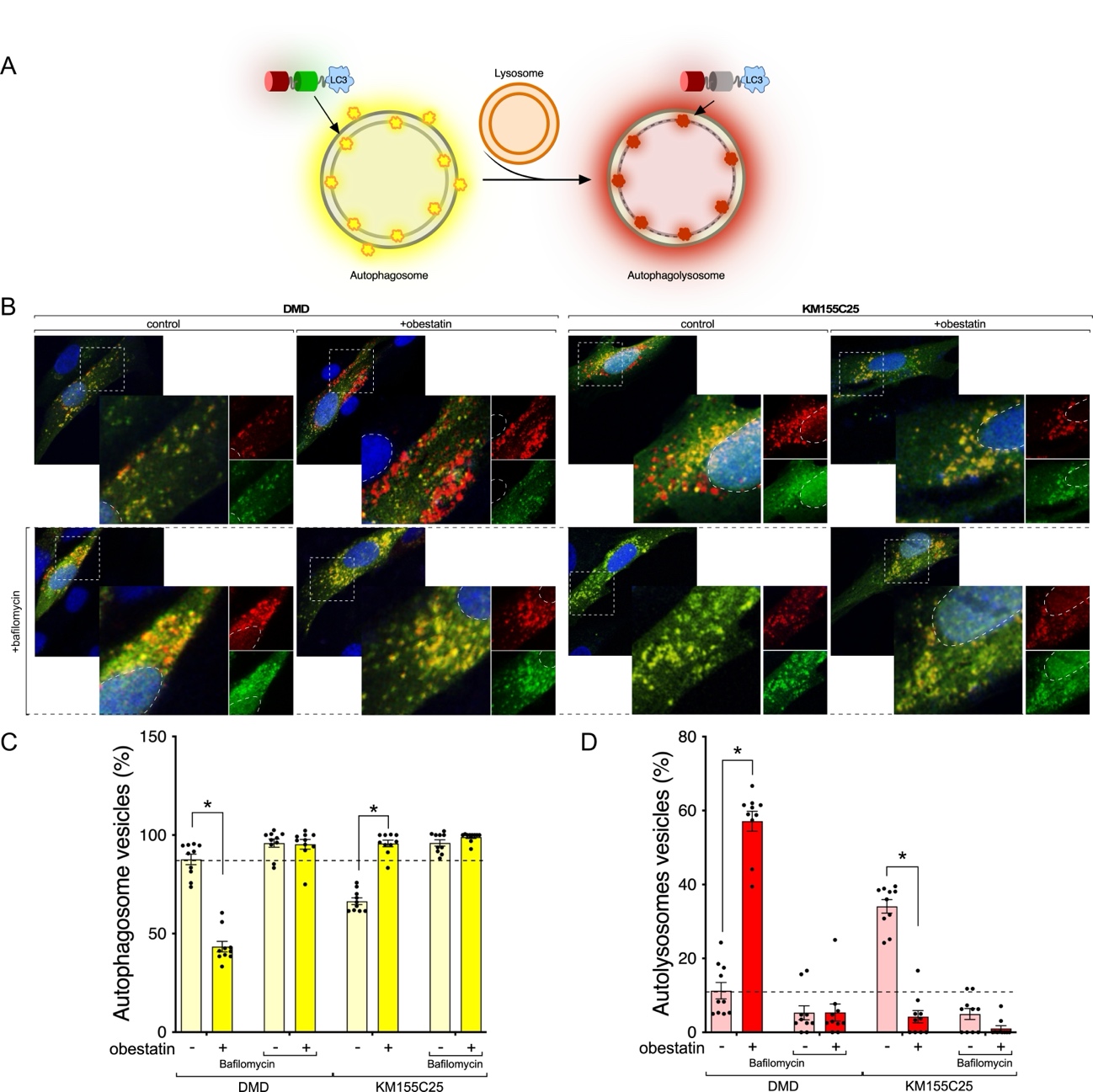
**

**FIGURE S3** (**A**) Schematic of mRFP-EGFP-LC3 reporter. When the reporter is associated to autophagosomes, both mRFP and EGFP fluoresce and autophagosomes are visualized as yellow puncta. Once the autophagosome fuses with a lysosome GFP fluorescence is quenched and autophagolysosomes appear as red fluorescent puncta. (**B**) Autophagy activity in DMD and KM155C25 myoblast cells expressing mRFP-GFP-LC3 treated with obestatin (10 nM) or PBS (control) for 24 h in the presence or absence of bafilomycin treatment. (**C**) Quantification of autophagosome vesicles. (**D**) Quantification of autolysosome vesicles. In C and D, insets show higher magnification and nuclei are highlighted with DAPI. Values are mean ± SEM (n= 50 cells from 6 independent experiments; *^*^* *p* < 0.05).
